# Evidence for a Fourteenth mtDNA-Encoded Protein in the Female-Transmitted mtDNA of Marine Mussels (Bivalvia: Mytilidae)

**DOI:** 10.1371/journal.pone.0019365

**Published:** 2011-04-27

**Authors:** Sophie Breton, Fabrizio Ghiselli, Marco Passamonti, Liliana Milani, Donald T. Stewart, Walter R. Hoeh

**Affiliations:** 1 Kent State University, Kent, Ohio, United States of America; 2 Department of Biologia Evoluzionistica Sperimentale, University of Bologna, Bologna, Italy; 3 Department of Biology, Acadia University, Wolfville, Nova Scotia, Canada; Virginia Tech Virginia, United States of America

## Abstract

**Background:**

A novel feature for animal mitochondrial genomes has been recently established: i.e., the presence of additional, lineage-specific, mtDNA-encoded proteins with functional significance. This feature has been observed in freshwater mussels with doubly uniparental inheritance of mtDNA (DUI). The latter unique system of mtDNA transmission, which also exists in some marine mussels and marine clams, is characterized by one mt genome inherited from the female parent (F mtDNA) and one mt genome inherited from the male parent (M mtDNA). In freshwater mussels, the novel mtDNA-encoded proteins have been shown to be mt genome-specific (i.e., one novel protein for F genomes and one novel protein for M genomes). It has been hypothesized that these novel, F- and M-specific, mtDNA-encoded proteins (and/or other F- and/or M-specific mtDNA sequences) could be responsible for the different modes of mtDNA transmission in bivalves but this remains to be demonstrated.

**Methodology/Principal Findings:**

We investigated all complete (or nearly complete) female- and male-transmitted marine mussel mtDNAs previously sequenced for the presence of ORFs that could have functional importance in these bivalves. Our results confirm the presence of a novel F genome-specific mt ORF, of significant length (>100aa) and located in the control region, that most likely has functional significance in marine mussels. The identification of this ORF in five *Mytilus* species suggests that it has been maintained in the mytilid lineage (subfamily Mytilinae) for ∼13 million years. Furthermore, this ORF likely has a homologue in the F mt genome of *Musculista senhousia*, a DUI-containing mytilid species in the subfamily Crenellinae. We present evidence supporting the functionality of this F-specific ORF at the transcriptional, amino acid and nucleotide levels.

**Conclusions/Significance:**

Our results offer support for the hypothesis that “novel F genome-specific mitochondrial genes” are involved in key biological functions in bivalve species with DUI.

## Introduction

Apart from the nucleus, mitochondria are the only known organelles with their own DNA in animal cells. Given the abundance of animal mitochondrial DNA (mtDNA) in animal tissues, strict maternal inheritance, the different evolutionary rates of its genes and the absence (or very low level) of recombination [1]–[3], this genome has come to be considered a reliable and robust marker for phylogenetic and population genetic studies as well as a model for the study of genome evolution [3], [4]. Comparative mitochondrial genomics has revealed that animal mtDNAs are very conserved in terms of gene content [4]. These small circular and typically intron-less molecules encode 2 ribosomal RNAs, 22 transfer RNAs and 13 protein subunits of the mitochondrial respiratory chain complexes and ATP synthase. The other subunits of the electron transport system (i.e., ∼65 subunits in mammals) as well as all the proteins and factors involved in other mitochondrial functions, such as mtDNA replication and mtDNA expression, are nuclear-encoded [4], [5]. However, with the increasing number of published mitochondrial genome sequences, examples of species that deviate from the gene content norm have been described in different animal groups including multiple species lacking one of the standard mitochondrial protein-coding genes [3]. In contrast, additional mitochondrial protein-coding genes, usually found in mtDNAs of the closest unicellular relatives of animals (e.g., *mutS*, *dnaB*, *atp9*, *tatC* in protists), have only been identified and annotated in the mitochondrial genomes of non-bilaterian animals (i.e., Cnidaria and Porifera; [3]).

One intriguing observation that emerged from sequencing studies of whole animal mtDNAs is the occurrence of numerous open reading frames (ORFs) of unknown function that are present in closely related species but for which homologues cannot be determined among more distantly related species [3], [6]–[11]. In bacterial and eukaryotic nuclear genomes, unique proteins that do not have recognizable homologues in other organisms (or that exist only in very closely related organisms) are commonly called “lineage-specific”, “taxonomically restricted” or “orphan” genes [12]–[14]. Interestingly, these lineage-specific genes have been shown to be involved in key biological functions and important adaptive processes [13]–[15]. For example, it has been demonstrated that species-specific differences in tentacle formation in the cnidarian genus *Hydra* correlate with expression of a taxonomically restricted gene encoding a small secreted protein of ∼85–105 amino acids [13]. Thus, the lineage-specific open reading frames that occur in animal mitochondrial genomes could potentially have functional significance. Lineage-specific mtDNA-encoded proteins are already known to play a role in sex determination in angiosperm plants exhibiting cytoplasmic male sterility [16]. Recently, we demonstrated the expression of two novel, sex-associated mtDNA-encoded proteins, the F- and M-*ORFs*, in freshwater mussels (Bivalvia: Unionoida) [11]. Although bioinformatics tools have not allowed us to characterize these genes, we have found that the female-transmitted ORF protein is not only present in mitochondria but, more surprisingly, it is also present on the nuclear membrane and in the nucleoplasm of eggs [17]. These results established novel features for animal mitochondrial genomes: the presence of additional, lineage-specific, mtDNA-encoded proteins with functional significance and the involvement of mtDNA-encoded proteins in extramitochondrial functions. Interestingly, this discovery has been made in the only known animal group that does not transmit its mtDNA exclusively maternally [18], [19].

Unlike the system of strict maternal inheritance described in other animal species, bivalves belonging to the orders Mytiloida, Veneroida and Unionoida instead possess a system of doubly uniparental inheritance (DUI) of mtDNA [18], [19]. Specifically, DUI is a “mother-to-daughter” and “father-to-son” mtDNA inheritance system where females transmit their mt genomes (F mtDNA) to both sons and daughters, and males transmit their mt genomes (M mtDNA) to their sons [20]–[23]. Female offspring are typically homoplasmic ( = containing one mt genome) and male offspring are heteroplasmic [21], [23] but see [24], [25]. In marine mussels *Mytilus* spp., male somatic tissue contains predominantly the mtDNA of the mother (F genome) but male gametes contain exclusively the mtDNA of the father (M genome) [26], [27]. In contrast, the M-type genome is predominant in male somatic tissues of the marine clam *Venerupis philippinarum*, and also exclusive in male gametes [28]. Remarkably, amino acid sequence divergences between sex-associated mtDNAs can reach 20% (uncorrected p-distance) in marine mussels *Mytilus* and up to 50% in freshwater mussels [29]–[31]. The latter observation is likely due, in part, to the relative stability and antiquity of the unionoid bivalve F and M genomes, i.e., these two mt genomes have been separately transmitted for >200 my.

**Figure 1:**
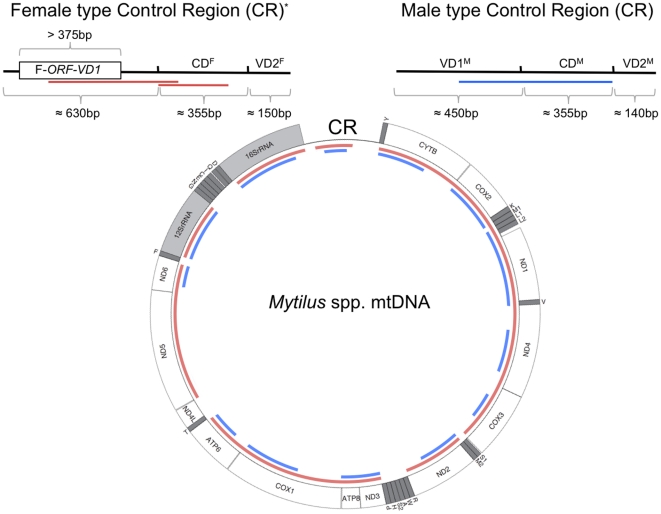
A typical *Mytilus* mitochondrial genome. All genes are encoded on the same strand. Gene identities: *nd1-6* and *nd4l*, NADH dehydrogenase subunits 1–6 and 4L; *cytb*, cytochrome b; *cox1-3*, cytochrome c oxidase subunits I–III; *atp6-8*, ATP synthase subunit 6 and 8 (protein-coding genes in white); 12SrRNA and 16SrRNA, small and large subunits of ribosomal RNA (in light gray). Transfer RNA genes are depicted by one-letter amino acid codes (in gray). The red and blue lines at the inner periphery of the ring represent EST sequences for the F and M mt genomes of *M. edulis/M. galloprovincialis*, respectively. Schematics of the structure of a typical F-type (left) and M-type (right) control regions, which are located between the 16SrRNA and trnY genes, are shown. The F-*ORF-VD1* is identified in the F-type control region. CR, control region; CD, conserved domain; VD1, variable domain 1; VD2, variable domain 2 [39], [40]. The mean size of each domain of the CR is shown. *The “standard” F-type CR of *M. trossulus*, which is a F/M recombinant CR, is not presented.

The newly identified F- and M-*ORFs* in freshwater mussels are not the only mitochondrial novelties with functional significance in these bivalves. It has been proposed that the C-terminus coding extension of the *COX2* protein (M*cox2e*), which is unique to freshwater mussel M genomes [32]–[36], could represent an “M-specific label” for sperm mitochondria that determines their fate in the fertilized eggs (as observed in *Mytilus* [37], [38]). In the marine mussel *Mytilus* spp., it has been proposed that the primary candidate for sequences that control the mode of inheritance of the two mitochondrial genomes would reside in the first variable domain (VD1) of the control region (CR) [39], [40]. The *Mytilus* CR can be divided in three domains based on indels and nucleotide variation [39], [40] (see also Figure 1): the first variable domain (VD1), which is the longest region of the CR, followed by a highly conserved middle domain (CD) and then a second variable domain (VD2), which is the shortest region of the CR. While the average DNA divergence between F and M genomes over the whole molecule may reach ∼20%, CD has diverged by only 1.5%, VD2 by about 15%, whereas VD1 is the most divergent part of the entire mt genome with DNA divergences averaging 50% [39]–[41]. Clearly, VD1 is under different, potentially sex-specific selective constraints, suggesting that it could play different roles in the F and M genomes [39], [40].

An interesting difference between marine mussels and freshwater mussels is that only in the former group have F mt genomes periodically experienced “role-reversal events” and invaded the male route of inheritance, resulting in the formation of new M mt genomes [42]–[44]. These new M mt genomes (known as recently masculinized M types) are essentially recombinants composed of an F genome's coding and control regions with an additional CR from a so-called “standard M” genome inserted into the F-type CR [30], [45]–[47]. It has therefore been suggested that the incorporation of sequences from the CR of a standard M genome into an F genome could be responsible for the paternal transmission route of the recombinant mtDNA genome [18], [30], [39], [47], [48]. However, attempts to confirm mitochondrial sequences and/or mt encoded proteins that are responsible for the different modes of mtDNA transmission under DUI have not been successful.

To date, much of the work on marine mussels, *Mytilus* spp., has focused on F vs. M-specific mtDNA motifs in the CR as potential features that could determine whether a genome will follow the maternal or the paternal mode of inheritance [39], [40], [45], [46]. Herein, we re-investigated all complete (or nearly complete) F, standard M and recently masculinized M mytilid mt genomes and control regions previously sequenced for the presence of ORFs that could have functional importance in these bivalves. Our results confirm the presence of a novel F genome-specific ORF of significant length (>375 nt), located in VD1, that most likely has functional significance in marine mussels (*Mytilus* spp.). The identification of this ORF in the relatively closely related *Mytilus edulis*, *M. galloprovincialis* and *M. trossulus* and in the more distantly related, also DUI-containing, *M. californianus* [49] and *M. coruscus* (evidence from GenBank) suggests that it has been maintained in the mytilid lineage (subfamily Mytilinae) for ∼13 million years [50]. Furthermore, this ORF likely has a homologue in the F mt genome of *Musculista senhousia*, a DUI-containing mytilid species in the subfamily Crenellinae [51]. Our results offer support for the hypothesis that “novel mt genome-specific genes” are involved in key biological functions, such as mtDNA transmission, in bivalve species with DUI.

## Results and Discussion

### Identification of open reading frames (ORFs) in the control regions of *Mytilus* mt genomes

To assess whether F and M mitochondrial control regions (CR) could possess ORFs that could have functional importance in *Mytilus* bivalves, we first investigated complete mtDNAs previously sequenced for the *Mytilus edulis* species complex (i.e., *M. edulis*, *M. galloprovincialis* and *M. trossulus* of the subfamily Mytilinae). Our results indicate that the VD1 of the F-type CR contains one conserved ORF (F-*orf-vd1*) of substantial length (>100aa). F-*orf-vd1* has complete start and stop codons and is located on the same strand as all other mtDNA-encoded genes (Figure 1 and Tables 1 and 2). The predicted length of the F-*ORF-VD1* protein is 163 amino acids (aa) for *M. edulis* and *M. galloprovincialis* and 153aa for *M. trossulus*. Interestingly, F-*ORF-VD1* was also detected in the F-type CR sequences of the more distantly related species *M. californianus* (129aa) and *M. coruscus* (127aa). These results are consistent with the hypothesis that the F-*ORF-VD1* region represents a new *Mytilus* mitochondrial gene with biological significance. In contrast to the F-type CR, conserved ORFs of similar sizes were not found in any of the M-type VD1 regions (Figure 1 and Tables 1 and 2). Assessing homology using a combination of sequence and position similarity, M-type ORFs of 94aa and 112aa were found in *M. edulis* VD1, ORFs of 21 and 42aa were found in *M. galloprovincialis* and ORFs of 73aa and 74aa were found in *M. trossulus* VD1 (Tables 1 and 2). Notably, ORFs of different sizes (24aa to >100aa) were identified within each species in the other complete M-type VD1 sequences available in GenBank, including the more distantly related *M. californianus* (30 and 32aa) (Figure S1). Moreover, analysis of these M-type ORF sequences using the testcode algorithm [52], which recognizes potential protein-coding sequences by evaluating the distribution of nucleotides at the third codon positions within a reading frame, suggests that they are non-coding (Table 3; probability of coding <30%). The gene-finding program Glimmer3 [53], which uses an interpolated Markov model scoring algorithm that computes the log-likelihood that a given interval on a DNA sequence was generated by a model of coding versus non-coding DNA, also failed to identify these M-type ORFs as putative protein-coding genes (data not shown). Contrary to the testcode algorithm that does not provide reliable results for sequences <200 bp [52], Glimmer3 is highly precise and sensitive to find protein-coding genes as small as 90 bp and usually detects >98% of genes in prokaryotic genomes with a limited number of false positive predictions [53]. It is also a very effective gene finder for eukaryotic genomes [54], and its accuracy to identify unannotated genes has been convincingly demonstrated by laboratory experiments [53]. For example, Glimmer3 predicted 16 out of 17 new proteins confirmed by protein-based experiments on the archaeon *Pyrococcus furiosus* [53]. Only the 13 typical mitochondrial protein-coding genes were successfully identified in the *Mytilus* M genomes using Glimmer3 (no additional ORF were found on the coding strand). These results support previous inferences that the M-type VD1 might function at the DNA or RNA level because of the presence of potential tRNA-like secondary structures in this domain [39], [40].

**Table 1:** Complete mitochondrial genome sequences of mytilid species with DUI used in this study.

Species	Gender	Genome Size (bp)	Position of the novel ORF	GenBank Accession Number	Reference
**MYTILOIDA**					
**Mytilinae**					
*Mytilus edulis*	F	16,740	1610..1942^c^	AY484747	[59], [60]
*Mytilus edulis*	M	16,622	1303..1641	AY823623	[30]
*Mytilus edulis*	M	16,624	1348..1632	AY823624	[30]
*Mytilus galloprovincialis*	F	16,744	13..504	AY497292	[29]
*Mytilus galloprocinvialis*	M	16,626	41..169 173..238	AY363687	[29]
*Mytilus galloprovincialis*	RM*	19,109	13..432 (F-like)	DQ399833	[47]
*Mytilus trossulus*	F^a^	16,781	13..540	DQ198231	[96]
*Mytilus trossulus*	M^a^	16,634	62..412	DQ198225	[96]
*Mytilus trossulus*	F^b^	18,653	443..904	GU936625	[97]
*Mytilus trossulus*	M^b^	16,578	16487..133	GU936626	[97]
*Mytilus trossulus*	M^b^	17,538	17465..148	GU936627	[97]
*Mytilus trossulus*	RM^b^,*	18,652	3248..3709 (F-like)	AY823625	[30]
**Crenellinae**					
*Musculista senhousia*	F	21,557	779..1144	GU001953	Passamonti et al. submitted
*Musculista senhousia*	M	21,612	-	GU001954	Passamonti et al. submitted

Note.—F = Female genomes; M = Male genomes; RM* = These genomes are recently-masculinized genomes (see text for details).

a These complete mtDNAs from Baltic *M. trossulus* derive throught recent introgression from *M. edulis* [96].

b These complete *M. trossulus* mtDNAs correspond to or have high affinity with the corresponding ancestral *M. trossulus* genomes from North America [30], [97].

**Table 2:** General Characteristics of F and M ORFs in the VD1 domain of the *Mytilus* spp.

Species	*orf-vd1*				*cox1*	*atp8*
	Length	A+T Content	Initiation and Termination codon	F/M divergence	F/M divergence	F/M divergence
*M. edulis* F	163 aa	52.4%	ATG – TAA	∼55%	18.4%	30.9%
*M. edulis* M	94 or 112 aa	∼65%	ATT – TAA			
*M. galloprovincialis* F	163 aa	51.8%	ATG – TAA	∼50%	18%	33.9%
*M. galloprovincialis* M	21 or 42 aa	∼65%	TTG – TAA			
*M. trossulus* F^a^	153 aa	53.5%	ATG – TAA	∼51%	18%	30.5%
*M. trossulus* M^a^	73 or 74 aa	∼62%	ATA – TAA			
*M. californianus* F	129 aa	57.2%	ATG – TAG	∼53%	20.5%^b^	-
*M. californianus* M	30 or 32 aa	∼68%	ATA – TAA			
*M. coruscus* F	127 aa	56.5%	ATG – TAA	-	-	-
*M. trossulus* RM^a^,*	FVD1 153 aa	53.7%	ATG – TAA	51%	-	-
	MVD1 71 aa	58.8%	GTG – TAA			
*M. galloprovincialis* RM*	FVD1 131 aa	51.2%	TTG – TAG	51%	-	-
	MVD1 67 aa	65.2%	ATA – TAA			
*M. senhousia* F	121aa	68.0%	ATC – TAA	-	17.6%	31.0%
*M. senhousia* M	-	-	-			

Note.— F = Female genomes; M = Male genomes; RM* = These genomes are recently-masculinized genomes (see text for details).

a These complete *M. trossulus* mtDNAs correspond to or have high affinity with the corresponding ancestral *M. trossulus* genomes from North America [30], [97].

b DNA divergences based on partial *cox1* sequences [42]. Sequence divergences are given in percentages nucleotide difference for the total number of aligned nucleotides.

CR and of the F-specific ORF in the unassigned region UR2 of *Musculista senhousia*.

**Table 3:** Testcode results for *Mytilus* spp.

Species/Gene	Testcode score	Probability of coding
F-*orf-vd1 M. edulis*	1.138	98%
F-*orf-vd1 M. galloprovincialis*	1.138	98%
F-*orf-vd1 M. trossulus*	1.011	92%
F-*orf-vd1 M. californianus*	0.856	77%
F-*orf-vd1 M. coruscus*	0.682	30%
F-*orf M. senhousia*	0.939	77%
“M-*orf-vd1*” *M. edulis*	0.542–0.636	7%
“M-*orf-vd1*” *M. galloprovincialis*	0.404–0.738	0%–30%
“M-*orf-vd1*” *M. trossulus*	0.403–0.516	0%–4%
“M-*orf-vd1*” *M. californianus*	0.586–0.597	7%

F-*orf-vd1* and “M-*orf-vd1*” and for *Musculista senhousia* F-*orf*.

The situation for the F-type VD1 is remarkably different. Specifically, “full length” F-*ORF-VD1*s were found in all *M. californianus* VD1 (n = 3), in all but 3 (12/15) *M. trossulus* VD1 and in all but 8 (41/49) of the *M. edulis* and *M. galloprovincialis* VD1 that have been completely sequenced to date (Figure S2) [39], [40], [45], [46], [55]–[57]. It is worth noting, however, that all three “truncated” vs. “full-length” *M. trossulus* F-*ORF-VD1*s (i.e., those with109aa instead of 153aa) and 7 of the 8 truncated *M. edulis/M. galloprovincialis* F-*ORF-VD1* (84 to 144aa instead of 163aa) were found in recombinant CR sequences that consist of both F-type and M-type CR segments or in duplicated F-type CRs [40], [46], [56]. In *M. trossulus*, all three truncated ORF sequences were due to a guanine base deletion at position 295 (out of 462 nt) in a segment consisting of a stretch of 5 Gs in “full length” *M. trossulus* F-*orf-vd1* sequences (Figure S2). However, this deletion was absent in all partially sequenced *M. trossulus* F-type VD1 available in GenBank (n = 156), which correspond to the first 407 nt of the F-*orf-vd1* and, when translated, to the first 135aa of the F-*ORF-VD1* without any stop codon. These observations raise the possibility that the 3 truncated *M. trossulus* F-*orf-vd1* sequences might represent sequencing errors. In the case of *M. edulis/M. galloprovincialis*, all “truncated” F-*orf-vd1* sequences observed in recombinant CR sequences were also found to occur only in sperm, i.e., these haplotypes were consistently absent from females [46], suggesting that they could represent recently-masculinized CR sequences (see below). A unique “truncated” F-*orf-vd1* sequence, due to a nucleotide insertion in a non-recombinant CR sequence, has been found in the complete *M. edulis* F genome (Figure S2), which was obtained from cloning experiments [58]–[60]. Multiple rounds of cloning or a sequencing error could explain this particular exception.

### Support for identifying F*-orf-vd1* as a protein-coding gene

#### Support at the transcriptional level

The maintenance of “full length” F-*orf-vd1* regions in the closely related *Mytilus edulis*, *M. galloprovincialis* and *M. trossulus* as well as in the more distantly related *M. californianus* and *M. coruscus*, which represents ∼13 million years of *Mytilus* mussel evolution [50], strongly argues in favor of functionality for this open reading frame. The hypothesis that the F-type but not the M-type VD1 encodes a protein is consistent with previous observations that F and M VD1 are under different selective regimes and likely explains why the intergenomic DNA divergences between F and M VD1 are the highest for *Mytilus* mitochondrial genomes [39], [40]. As listed in Table 2, the mean DNA divergences between aligned portions of the putative F-*orf-vd1* and the M-type VD1 within each species exceed by far those observed for the recently identified, rapidly evolving *atp8* gene [61]. The maintenance of a functional ORF only in the F lineage would explain not only the high intergenomic divergences but also support the hypothesis that VD1 has a sex-specific function [39], [40], [45], specifically, that the F-*ORF-VD1* is a novel mitochondrial protein with a F-specific function in *Mytilus* mussels. In support for such a role, testcode predictions of protein coding function for the F-*orf-vd1*sequences are, with the exception of *M. coruscus* (probability of coding = 30%), all very high (Table 3). The gene-finding program Glimmer3 [53] also predicts the protein coding nature of the F-*orf-vd1* sequences. The program attributes a score to each orf, providing a consistent scale to compare coding potential scores of different orfs [53]. For example, the *Mytilus edulis* F-*orf-vd1* presents a higher coding potential score (8.99) than the typical mitochondrial protein-coding genes *cox1-cox2-cox3*, *cob*, *nd3-nd4l*, and *atp8* (3.93 to 8.26), and a lower score than *nd1-nd2-nd4-nd5-nd6*, and *atp6* (9.23 to 10.77). The reason why testcode classified the *M. coruscus* F-*orf-vd1* sequence as non-coding and the *M. californianus* and *M. senhousia* F-*orfs* as having 77% probability of coding could be explained by the high variability of this putative gene (see below). Indeed, fast-evolving genes are often rated as non-coding by the testcode algorithm, presumably because the mechanisms generating diversity are stronger than the ones encouraging consistent codon preference [52].

Interestingly, corroborative evidence for the protein-coding nature of F-*orf-vd1* was also obtained from BLASTN searches against dbEST (Expressed Sequence Tag division, EST_others). For example, for the more extensively studied *M.edulis/M. galloprovincialis*, a total of 366 and 194 ESTs were aligned to the complete F and M mt genomes with nucleotide identity >96%, respectively (out of 24,611 ESTs from >20 different polyadenylated cDNA libraries; [62]–[66]). The ESTs cover 14,994 bp (89.6%) of the F mt genome and 9,868 bp (59.3%) of the M mt genome. Figure 1 reports the *M. edulis/M. galloprovincialis* F and M ESTs mapped on the completely sequenced *Mytilus* mtDNA. The majority of ESTs (n = 168 for the F genome and n = 156 for the M genome) are derived from 16S and 12S rRNAs, suggesting a higher expression level and/or a higher stability compared to other mt genes. Given that ESTs come from multiple cDNA libraries constructed using different methods [62]–[66], and because it is absent in both F and M mt genomes and thus less likely to be the result of the trimming of low quality sequences or the cloning procedure (e.g., [66], [67]), the lack of ESTs corresponding to the F- and M-type *nad4L* gene suggests that this transcript might be expressed at low levels and/or might be rapidly degraded in *M. edulis/M. galloprovincialis*. Similarly, the absence of several tRNA-like ESTs for both F and M genomes could be explained by their removal from mature polyadenylated transcripts [67], [68]. Furthermore, the typical utilization of *Mytilus* somatic tissues in cDNA library preparations [62]–[66] can explain the lower coverage of the M mt genome by M-type ESTs, given that somatic tissues predominantly contain and express the F-type mtDNA [26].

Remarkably, the EST analysis yielded 11 significant hits for *M. edulis* and *M. galloprovincialis* F-*orf-vd1* sequences whereas neither the second variable domain (VD2) of the F-type control region nor the M-type VD2 was present in ESTs (Figure 1; the 11 hits are AJ626121, AJ626242, AJ626130, AJ626120, AJ626205, AJ626443, AJ626444, AJ626129, AJ626131, AJ623360, AJ624518). Because only the last 270 bp of F-*orf-vd1* are covered by EST sequences (Figure 1), one could argue that these transcripts represent by-products from an unprocessed polycistronic transcript precursor. However, the observation that 6 other mitochondrial genes (i.e., *cox1*, *cob*, *nad1*, *nad2*, *nad4*, and *nad5*) were not represented by “full-length ESTs” containing the entire gene, i.e. only partial transcripts were found in dbEST (data not shown), suggests a reduced enrichment in full-length cDNAs in *Mytilus* libraries. Moreover, since the VD1-like ESTs have been obtained by oligo-dT priming of mussel mRNA [64], [65], they are expected to originate from polyadenylated mature transcripts. Taking together, these findings suggest that the F-type VD1 is expressed in the *Mytilus* mitochondrial proteome. On the other hand, since we also found significant hits for F-type CD (3 hits) and part of M-type VD1 and CD sequences (2 hits) (see Figure 1), the possibility remains that all of these polyadelynated transcripts function at the RNA level. Polyadenylated transcripts derived from a putative non-coding region have previously been reported in the oyster *Crassostrea gigas* [67]. The authors hypothesized that this intergenic segment located between the *atp6* and *nd2* genes could represent the mitochondrial control region, which is polyadenylated at a high level in several mammal species [67]–[70]. However, it appears that this intergenic segment in *C. gigas* actually contains the “formerly reported as missing” *atp8* gene in these bivalves [61]. Interestingly, the polyadenylated mitochondrial CR sequences observed in mammals have been proposed to be multi-functional molecules serving as primers for mtDNA replication, regulators for replication and translation processes through rRNA binding as well as protein-coding mRNAs [68]–[73]. For example, Nakamichi et al. [73] reported a CR transcript in humans that could code for a peptide of 76 amino acids. The possibility thus remains that CR transcripts in *Mytilus* function in different ways, i.e., that F-*orf-vd1* functions at both RNA and protein levels (or at the protein level only) and that other CR transcripts function at the RNA level. Another hypothesis would be that the M-type VD1 also functions at the protein level but that its function is supported by smaller ORFs such as those found in the M-type VD1 domain of *M. californianus*. Further data collection and investigation will be essential to clarify the functional role of these CR transcripts and ORFs.

#### Support at the amino acid level

The analysis of the taxonomic distribution of the F-*ORF-VD1* in mytilid mussels is one other important step in the assessment of its potential functional role as a protein. To establish whether the F-*ORF-VD1* is taxonomically restricted to the genus *Mytilus* or if it is an evolutionary feature of mytilid mussels, we screened for the presence of F-specific ORFs in the newly sequenced mitochondrial genome of the DUI-containing mytilid *Musculista senhousia* from the subfamily Crenellinae (Passamonti et al. submitted). Within the Bivalvia, mytilid mussels form a monophyletic group where *Musculista* and *Mytilus* are invariably clustered together, while freshwater mussels (Unionoida), including the species *Venustaconcha ellipsiformis*, are confirmed basal and fully separated from all other autolamellibranchiate lineages, including the Mytilidae ([31], [74]; Plazzi & Passamonti unpublished). Interestingly, we found one relatively large unassigned region specific to the F genome of *M. senhousia* (UR2 = 543 bp) preceding the control region and containing an ORF of considerable length (121aa). The *M. senhousia* F-*ORF* possesses complete start and stop codons, is located on the same strand as all other mtDNA-encoded genes, and has a probability of coding of 77% (Tables 2 and 3).

**Figure 2:**
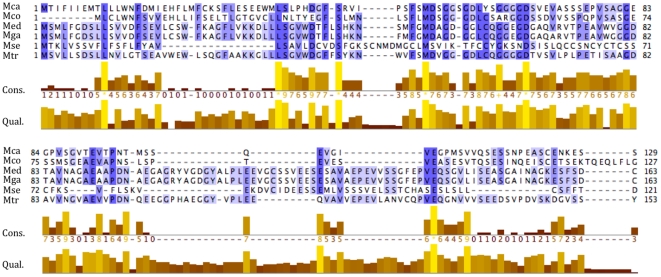
T-COFFEE alignment of the translated F-specific ORF sequences of mytilid mussels. Three of more identical amino acids within a column are highlighted in blue. Conservation (Cons.) score values and quality (Qual.) of the alignment are indicated. Dashes (–) denote a missing residue at this position in comparison with other sequence(s). Mca, *M. californianus*; Mco, *M. coruscus*; Med, *M. edulis*; Mga, *M. galloprovincialis*; Mse, *M. senhousia*; Mtr, *M. trossulus*.

**Figure 3:**
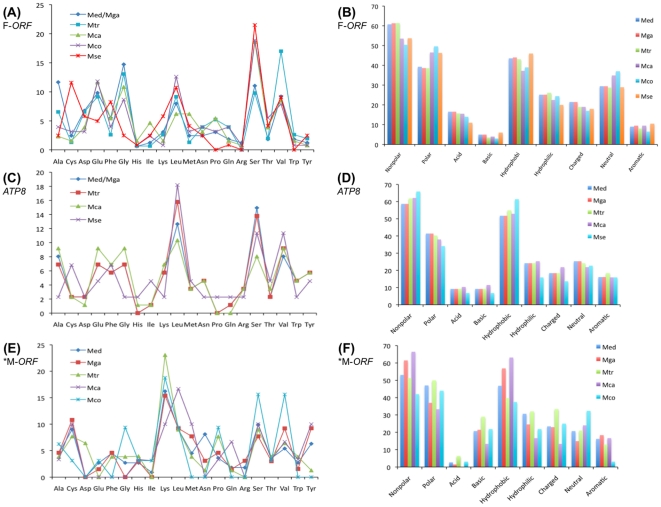
Amino acid composition of mytilid F-specific ORFs, protein-coding gene *ATP8* and putative M-*ORF-VD1*. (A) Overall amino acid composition of *Mytilus* spp. F-*ORF-VD1* and *M. senhousia* F-*ORF* protein sequences. (B) Composition of chemically equivalent amino acids of *Mytilus* spp. F-*ORF-VD1* and *M. senhousia* F-*ORF* protein sequences. (C) Overall amino acid composition of *Mytilus* spp. F-*ATP8* and *M. senhousia* F-*ATP8* protein sequences. (D) Composition of chemically equivalent amino acids of *Mytilus* spp. F-*ATP8* and *M. senhousia* F-*ATP8* protein sequences (*M. coruscus atp8* sequence is not available in GenBank). (E) Overall amino acid composition of the putative *Mytilus* spp. M-*ORF-VD1* (*mean values for several M-*ORF-VD1* GenBank sequences for each species). (F) Composition of chemically equivalent amino acids of the putative *Mytilus* spp. M-*ORF-VD1* (such ORF has not been found in *M. senhousia*). Amino acid composition is reported as percentage. Mca, *M. californianus*; Mco, *M. coruscus*; Med, *M. edulis*; Mga, *M. galloprovincialis*; Mse, *M. senhousia*; Mtr, *M. trossulus*.

**Figure 4:**
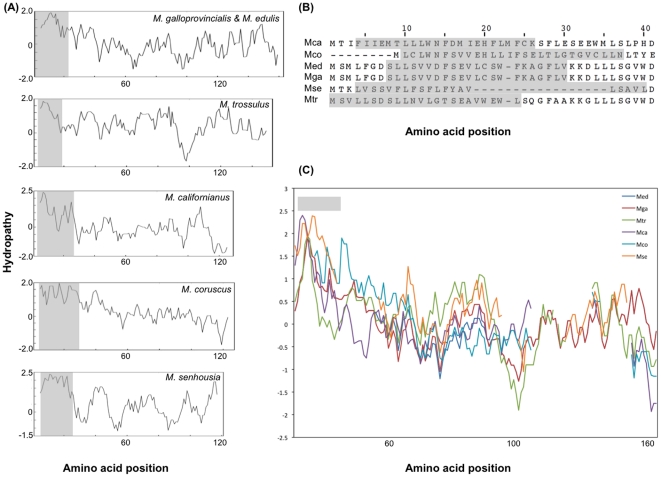
Comparisons of *Mytilus* spp. F-*ORF-VD1* and *M. senhousia* F-*ORF* hydropathy profiles. (A) Profiles for each species were calculated by the method of Kyte and Doolittle [104]. Numbers below profiles designate amino acid positions in each protein. Predicted transmembrane domains according to TMpred [102] (all with significant scores >500) are shown in light gray (It has to be noted that a single TMH in the N terminal portion of each F-*ORF* proteins has also been identified using HMMTOP [101]). (B) T-COFFEE alignment of the translated N terminal portions of mytilid F-specific ORF sequences. Predicted transmembrane domains according to TMpred [102] are shown in light gray. (C) Kyte and Doolittle profiles for the F-*ORF* protein alignment with the homologous amino acid sites in the same position on the x axis. The predicted transmembrane domain according to TMpred [102] is shown in light gray. Mca, *M. californianus*; Mco, *M. coruscus*; Med, *M. edulis*; Mga, *M. galloprovincialis*; Mse, *M. senhousia*; Mtr, *M. trossulus*.

At the amino acid level, comparisons among predicted sequences for *Mytilus* spp. F-*ORF-VD1* and the *M. senhousia* F-*ORF* revealed this putative gene as the least conserved in the *Mytilus* F lineage, with aa sequence identities ∼1.5–2.5 times lower than those obtained for the highly variable *ATP8* protein, and among the least conserved in mytilid mussels (Table 4). For example, 24% amino acid identities are observed for the F-specific ORFs between the distantly related *Mytilus edulis* and *Musculista senhousia* species, whereas 18% amino acid identities are observed for *ATP8* (and 74% for *COX1*). Figure 2 shows the alignment of all mytilid F-specific ORFs. The greatest similarity among all species is principally found within a stretch of 60 residues in the middle of the protein sequence. As expected for a rapidly evolving protein, sequence differences between the F-*ORF* from *M. senhousia* and the F-*ORF-VD1* from *Mytilus* spp. are more pronounced (Figure 2). These results suggest that, if they are functionally equivalent proteins, constraints on *M. senhousia* F-*ORF* and *Mytilus* F-*ORF-VD1* are imposed at higher levels of protein structure rather than the amino acid sequence level. In support of this hypothesis, the amino acid compositions of the *M. senhousia* F-*ORF* and *Mytilus* F-*ORF-VD1* are slightly different (Figure 3A), but their compositions of chemically equivalent amino acids (i.e., with similar properties) are similar (Figure 3B) and comparable to what is observed for the fast-evolving mtDNA-encoded protein *ATP8* (Figure 3C & D). In contrast, the translated M-*ORF* found in the VD1 domain of M genomes shows high variability with regards to amino acid composition (Figure 3E & F). These results, which can be explained by the presence of frameshifts, premature stop codons and important differences in length in M-*ORF* sequences, are in agreement with the hypothesis that only the mytilid F-*ORF* codes for a functional protein. If the F-*ORF* would not be functional at the protein level, one would expect the presence of within- and between-species “coding disablements”, such as the numerous frameshift mutations and premature stop-codons seen in M-*ORF* sequences. Furthermore, amino acid compositional similarities among mytilid species (or even between F-*ORF* sequences and protein-coding genes within a same genome) would not be expected from non protein-coding sequences since they are not subject to selective pressure to preserve protein structure and function [75]. In this latter case, however, similar mtDNA nucleotide bias and common evolutionary history could at least partly explain the observed results [75]–[77]. For example, it has been demonstrated that amino acid frequencies in proteins or in translations of randomly selected non-coding sequences are changing in response to the genomic change in G+C (or A+T) content, that is GC-rich codons and corresponding amino acids will increase in frequency in proteins and translated non-coding sequences in genomes with increasing G+C content, whereas AT-rich codons and corresponding amino acids will increase in frequency in proteins and translated non-coding sequences in genomes with increasing A+T content [75], [76]. A+T contents are relatively similar for both F-*ORF-VD1* and *ATP8* in *Mytilus* spp. (52–57% for the F-*ORF-VD1* and 58–59% for *ATP8*), whereas *Musculista* values are higher with 68% A+T for the F-*ORF* and 68.9% for *ATP8*. Proportions of AT-rich codons and corresponding FYMINK amino acids [75] are slightly higher in *Musculista* for the F-*ORF* (25.6% vs. 13–21.7% for *Mytilus*) but comparable for *ATP8* (26% vs. 26–28.5% for *Mytilus*). Although preliminary, these results indicate a potential correlation between DNA composition and amino acid compositional similarities of F-*ORF* sequences but they do not rule out the hypothesized protein-coding function of the F-*ORF* in mytilid mussels. In addition and of significant relevance here is our observation that a single, conserved predicted transmembrane helix (TMH) is present in the N terminal portion of all mytilid F-*ORF* proteins (Figure 4), suggesting that this putative gene would assume the underlying DNA composition of the mtDNA to the extent that this does not interfere with the secondary structure and biochemical function of the protein. Like typical animal mitochondrial genes, which all encode TMH proteins of the oxidative phosphorylation system in the inner mitochondrial membrane [4], [5], the F-*ORF* protein could be an element of the electron transport chain or ATP synthase complex in mytilid mussels. However, the recent finding that F-*ORF* proteins likely play a role in sex determination in unionoid bivalves indicates that the mytilid F-*ORF* proteins could also have a non-oxidative phosphorylation function [17]. Although our results suggest stabilizing selection on the F-*ORF* region's amino acid composition and secondary structure and support the protein-coding hypothesis, further protein-based analyses will be necessary to characterize the biological significance of the mytilid ORFs, and to verify if they are functionally equivalent. Additional complete mt genomes, from mytilid and non-mytilid bivalves, are needed to elucidate the number, taxonomic distribution, and evolution of uncharacterized ORFs in this group of molluscs.

**Table 4:** Interspecies comparisons in the mytilid F lineage (*Mytilus* spp. and *Musculista senhousia*) for the fast evolving F-*ORF-VD1* & F-*ORF* and *ATP8* genes: % amino acid identities.

	Amino Acid Identities for F-*ORF-VD1*	Amino Acid Identities for *ATP8*
*M. edulis*/*M. galloprovincialis*	97.55%	95.35%
*M. edulis*/*M. trossulus*	45.40%	69.77%
*M. edulis*/*M. californianus*	27.69%	63.95%
*M. edulis*/*M. coruscus*	23.44%	-
*M. edulis*/*M. senhousia*	24.00%	18.18%
*M. galloprovincialis*/*M. trossulus*	45.40%	71.26%
*M. galloprovincialis*/*M. califonianus*	26.92%	65.52%
*M. galloprovincialis*/*M. coruscus*	20.47%	-
*M. galloprovincialis*/*M. senhousia*	24.00%	18.18%
*M. trossulus*/*M. californianus*	33.33%	62.22%
*M. trossulus*/*M. coruscus*	26.77%	18.18%
*M. trossulus*/*M. senhousia*	14.29%	-
*M. californianus*/*M. coruscus*	51.24%	-
*M. californianus*/*M. senhousia*	13.33%	22.73%
*M. coruscus*/*M. senhousia*	25.00%	-

Note.—No *ATP8* sequence is available for *M. coruscus*. Amino acid identities % are given for the total number of aligned amino acids.

#### Support at the nucleotide level

Due to alignment issues, nucleotide-level analyses were performed using the more similar *Myilus* spp. F-*orf-vd1* sequences (i.e., we excluded *Musculista senhousia*). Comparisons of synonymous substitutions per synonymous site (Ks), nonsynonymous substitutions per nonsynonymous site (Ka) and Ka/Ks ratios within and between *Mytilus* species also provide evidence that F-*orf-vd1* encodes a functional protein: within species Ka/Ks ratios are higher than between species Ka/Ks ratios, which are usually well below 1 and thus indicate purifying selection (Table 5). A lower between-species Ka/Ks is a common finding for mtDNA-encoded protein genes in animals and is explained by the elimination of mildly deleterious polymorphisms from populations before fixation [78]–[80]. Such results would not be expected for non-protein coding sequences. A more exhaustive analysis was undertaken to test the null hypothesis of neutrality and search for the signature of purifying and/or positive selection by calculating the Ka/Ks ratio at each codon site with the SELECTON program using a Bayesian approach [81]. In essence, neutrality is indicated by Ka/Ks = 1, purifying selection by Ka/Ks<1, and positive selection is usually invoked as a possible explanation for rare cases where the pattern Ka/Ks>1 is observed. The idea is that substitutions at synonymous sites are largely selectively neutral relative to the intensity of selection at nonsynonymous sites and very low proportion of amino acid replacement can be interpreted as a reflection of purifying selection maintaining a functional protein. By contrast, under positive selection, rapid replacement of an amino acid is advantageous to the organism; hence, nonsynonymous mutations are fixed at a rate higher than that of neutral synonymous ones [81], [82]. According to our results, the MEC selection model was significantly preferred than the null model M8a (AICc score for MEC is 8195.54 while AICc score for M8a is 13981.56) and suggested 35 putative positively selected residues. For the full alignment and Bayesian Ka/Ks ratios obtained from the models, see Figure S3. Specifically, *Mytilus* spp. *COX1* residues were all found to be under strong purifying selection whereas of the 165 amino acid positions in the F-*ORF-VD1* alignment portion, 125 residues (76%) were found under purifying selection, 5 (3%) were under neutral selection and 35 (21%) were found to possess a Ka/Ks>1, indicating positive selection. However, the inference of positive selection was not considered statistically significant for any particular residue. Most of the unconserved 35 positions were located in the C-terminal portion of the protein (54%), whereas 23% of them were found within the more conserved stretch of 60 residues in the middle of the protein sequence (see Figure 2) and 17% were found in the TMH portion of the *Mytilus* spp. F-*ORF-VD1* (data not shown). Overall, our results suggest that even if most of the sites are subjected to purifying selection, which is suggestive of a functional constraint, the presence of several sites with Ka/Ks>1 indicate that the F-*orf-vd1* is a fast evolving gene in the *Mytilus* F genome. As recently proposed for the highly variable *atp8* gene in these species [83], relaxed purifying selection coupled with the compensation-draft feedback process [80] could cause the faster evolution of F-*orf-vd1*. Specifically, the compensation-draft feedback process postulates that fixation of a mildly deleterious mutation favors compensatory mutations within the same or interacting polypeptides, which in turn can result in fixation of new mildly deleterious mutations by genetic draft due to the linked, non-recombining genes in mitochondrial DNA [80]. The compensation-draft feedback process could have been initiated by selection for an F-specific function for the F-*ORF-VD1*.

**Table 5:** DNA comparisons in the *Mytilus* F lineage for the fast evolving F-*orf-vd1* gene: selective pressures at synonymous and nonsynonymous sites.

	K_A_	K_S_	K_A_/K_S_
**Within species comparisons**			
*M. edulis*	0.007	0.010	0.7
*M. galloprovincialis*	0.006	0.008	0.75
*M. trossulus*	0.004	0.005	0.8
*M. californianus*	0.007	0.000	-
**Between species comparisons**			
*M. edulis*/*M. galloprovincialis*	0.011	0.012	0.916
*M. edulis*/*M. trossulus*	0.379	0.785	0.482
*M. edulis*/*M. californianus*	0.863	1.048	0.443
*M. edulis*/*M. coruscus*	0.840	2.112	0.397
*M. galloprovincialis*/*M. trossulus*	0.388	0.807	0.480
*M. galloprovincialis*/*M. californianus*	0.863	2.132	0.404
*M. galloprovincialis*/*M. coruscus*	0.840	2.125	0.395
*M. trossulus*/*M. californianus*	0.934	1.506	0.620
*M. trossulus*/*M. coruscus*	0.906	1.615	0.560
*M. californianus*/*M. coruscus*	0.330	0.788	0.418

Note.— The divergence in synonymous sites (K_S_) and the divergence in nonsynonymous sites (K_A_) have been calculated by the modified Nei-Gojobori method with Jukes-Cantor correction. Within species comparisons have been calculated using D-loop sequences from the following studies: [39], [40], [45], [46], [55]–[57]; Barna and Showman unpublished.

Interestingly, the newly discovered F and M lineage-specific proteins in freshwater mussel species are also among the fastest evolving proteins coded by freshwater mussel mitochondrial genomes [11], [17]. These findings suggest that mt lineage-specific genes or DNA regions are potential targets for positive selection and thus they might play an important role in bivalve speciation (i.e, mitochondrial populations of the same species could quickly diverge, and possibly become reproductively isolated because of mitochondrial-nuclear incompatibilities; [84]).

### Novel mtDNA-encoded genes in bivalve species with DUI

Assuming a single origin of DUI [31], [85], [86], the F lineage specific ORFs in both marine and freshwater mussels could represent homologous genes. However, as is the case for the novel, F- and M-specific, mtDNA-encoded proteins in freshwater mussels [11], [17], the precise function of the F-*ORF-VD1* protein in marine mussels remains unclear. In freshwater mussels, no significant amino acid sequence similarity with known proteins was found for the F-*ORF* using BLAST Tools, but the estimated tertiary structure of the F-*ORF* from the species *Venustaconcha ellipsiformis* is consistent with involvement of this novel mitochondrial protein in DNA replication and/or DNA binding [17]. In the present study, sequence similarity searches for *Mytilus* F-*ORF-VD1* using PSI-BLAST [87] against non-redundant protein sequences and SWISSPROT databases also failed to detect significant sequence similarity with known proteins. However, searching against the Protein Data Bank (PDB; [88]) revealed that the *M. edulis* and *M. galloprovincialis* F-*ORF-VD1* exhibit relatively weak sequence similarity (E-value of 0.004) to an archaebacterial DNA helicase, suggesting that it could be a DNA-binding protein involved in regulation of mitochondrial DNA replication and/or transcription as might be the case for the F-*ORF* in freshwater mussels. To our knowledge, helicase genes have never been reported in animal mitochondrial DNA [3], [89]. However, a putative helicase has been reported in the mitochondrial genome of the plant *Marchantia polymorpha* [90]. Moreover, the possibility of open reading frames in the mitochondrial control region playing a role in the replication and/or transcription process has been previously reported in some mammals [6] and *Paramecium* [91]. It is also worth noting that many of the “unusual” protein-coding genes discovered in invertebrate mitochondrial genomes contain amino acid patterns characteristic of interaction with DNA [3], [8], [92], [93]. However, at this moment, it is not possible to confirm the hypothesis that the F-specific ORFs in marine and freshwater mussels are homologous due to their highly divergent nature and incomplete knowledge regarding their phylogenetic distribution.

Irrespective of a common vs. independent origins for the F-specific ORFs in marine and freshwater mussels, there are at least three possibilities for their source: (i) a gene homologous to ancestral bacterial protein-coding genes, (ii) a duplicated and diverged mitochondrial gene or (iii) a transfer from the nucleus to the mitochondrion [93], [94]. Again, because of their relatively fast evolutionary rate, the F-specific ORF sequences have probably changed to such an extent that their historical antecedents are no longer recognizable at the aa sequence level. Based on currently available data, the F-*ORF* in freshwater mussels has persisted for >200 my [11], [17]. As is the case for F-*ORF-VD1* in *Mytilus* spp. and F-*ORF* in *M. senhousia*, the sequence similarity is low among distantly related freshwater mussels species, but selection has maintained at least one aspect of the secondary structure of the protein: one predicted TMH in the N-terminal portion of the protein [11], [17]. However, the amino acid divergences and differences in amino acid composition between the mytilid ORFs and the F-*ORF* protein of the freshwater mussel species *Venustaconcha ellipsiformis* are much more pronounced than between the *Mytilus* spp. F-*ORF-VD1* and the *M. senhousia* F-*ORF* (Figure S4). Again, further protein-based analyses will be necessary to characterize the biological significance and critically evaluate the hypothesized functional equivalence of the F-specific ORFs in bivalves.

### The study of masculinized genomes to identify sequences responsible for mitochondrial transmission mode

As mentioned earlier, a phenomenon that characterizes marine mussels is that female-transmitted mt genomes have periodically experienced “role-reversal events” and invaded the male route of inheritance, resulting in the formation of new M mt genomes [42]–[44]. Previous sequencing studies have demonstrated that all “recently-masculinized” or RM-mitotypes examined to date in mytilid mussels are recombinants composed of an F genome's genes and CR plus an additional M-type CR [30], [45]–[47]. Consequently, it has been hypothesized that recombination with the introduction of a “Standard Male” or SM-type CR into an otherwise female type mt genome could be the first step in the masculinization process [48]. However, to establish that a genome is masculinized, one needs to demonstrate that the genome is the exclusive mtDNA molecule in the sperm of the male from which it was extracted [40], [47]. Indeed, RM-type sequences obtained from male gonad DNA extractions could be artifacts due to somatic tissue contamination. This logic makes the fully sequenced “C genome” of *M. galloprovincialis*, which was extracted from spermatozoa that were forced to swim through a Percoll™ solution to remove of any debris from somatic cells, the only verified masculinized genome sequenced to date [47]. We will thus mainly refer to this sequence in the section below.

**Figure 5:**
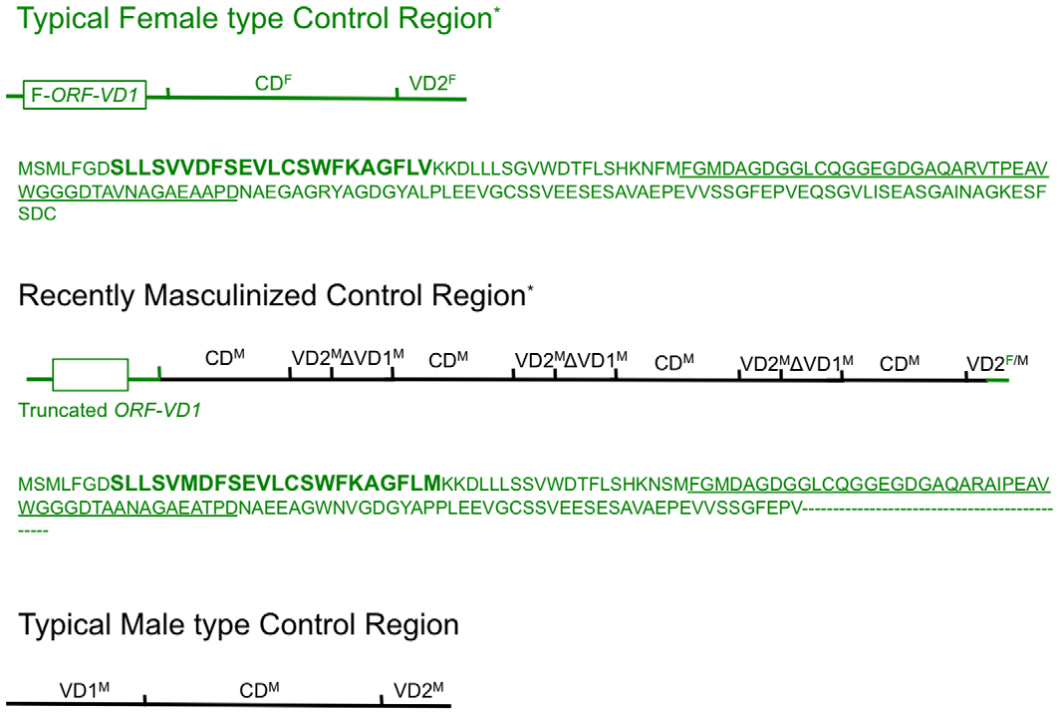
Schematics of the structure of a typical F-type (above), recently-masculinized (center, according to Venetis et al. [**47**]) and typical M-type (below) control regions. The F-*ORF-VD1* is identified in the F-type control region. The amino acids that constitute the putative transmembrane helix are indicated in boldface type and bigger characters. The stretch of ∼60 residues showing the greatest similarity among the species is underlined. Dashes (–) denote the missing amino acid residues in the truncated F-*ORF-VD1*. CD, conserved domain; VD1, variable domain 1; VD2, variable domain 2 [39], [40]. *The “standard” F-type CR of *M. trossulus*, which is a F/M recombinant CR, is not presented.

Because the first variable domain VD1 has been identified as the most likely site for sequences that could control the mode of inheritance of the mitochondrial genome [39], [40], examination of RM-type VD1 sequences is essential to address the hypothesis that these sequences could determine maternal vs. paternal inheritance. The control region of the recently masculinized, male-transmitted “C genome” of *M. galloprovincialis* is composed of an F-type VD1 followed by an M-type CD, an M-type VD2 and a truncated M-type VD1 (i.e., VD1^F^/CD^M^/VD2^M^/ΔVD1^M^) [40], [47]. After VD1^F^, the segment “CD^M^/VD2^M^/ΔVD1^M^” is repeated tandemly three times. The third repeat unit is followed by one complete CD^M^ and one F/M recombinant VD2 (Figure 5).

From an “ORF point of view”, the *M. galloprovincialis* “C genome” is different from the standard F-type mtDNA of the same species in having a truncated (at the C-terminus) F-*ORF-VD1* protein (139aa instead of 163aa; Figure 5). It has to be noted that the deleted protein region does not involve the conserved 60aa stretch observed among mytilid species. We have shown that standard F genomes consistently possess a “full length” F-*ORF-VD1* (with one exception that could represent a sequencing error), suggesting the hypothesis that a “full length” F-*ORF-VD1* is (i) necessary for maternal transmission and/or (ii) its presence could interfere with male transmission. Further evidence in support of this hypothesis is that, except for the singular sequence mentioned above, all truncated F-*ORF-VD1* sequences have been found in recombinant CR sequences and are consistently absent from females (i.e., these haplotypes occur only in sperm and could represent recently masculinized CR sequences). The presence of a truncated F-*ORF-VD1* in recently masculinized genomes thus suggests that, to enable paternal transmission, a standard F genome must gain a new M functionality (i.e., gain M-type CR sequences) as well as lose an F functionality (i.e., the disruption of F-*ORF-VD1*). One hypothesis is that the disruption of F-*ORF-VD1* occurs first and subsequently this disruption would facilitate F/M recombination, which has been proposed as the first step in the masculinization process [48]. An alternative hypothesis would be that the deletions in the F-*ORF-VD1* regions of RM genomes are a consequence of masculinization. If the F-*ORF-VD1* is required only for F mt function, its presence in a paternally-transmitted M genome would allow its degeneration. However, we still do not know enough about the recombination process and developmental genetics of DUI to speculate on where and when recombination is likely occurring, though it might happen during spermatogenesis when the five so-called “mega-mitochondria” form in the mid-piece of a spermatozoon by fusion of several smaller mitochondria [37], [95]. Because of the apparently dynamic nature of DUI in marine mussels, the “Standard Male” genomes in all *Mytilus* examined to date are likely the product of previous role reversal events. To reject the hypothesized primacy of F-*ORF-VD1* disruption in the masculinization process, one would need to look for paternally transmitted mt genomes containing a complete F-*ORF-VD1*. The data available to date, however, show that F-*ORF-VD1* is eventually lost in “Standard Male” genomes, reinforcing the hypothesis that this gene has an F genome-specific function.

Although speculative, we propose that F-*ORF-VD1* has been maintained in mytilid mitochondrial CRs to participate in the regulation of mt transmission and/or the regulation of F genome replication and transcription. This hypothesis is consistent with the recent suggestions that the RM-type genome sequenced for *Mytilus trossulus*, which was inferred to be a recently masculinized genome because of its extraction from a male gonad [30], would be in fact the F genome of *M. trossulus* [40], [83]. This particular genome indeed contains a complete F-*ORF*-VD1, which is consistent with the hypothesis of maternal transmission. However, further data collection and analyses will be essential to clarify the functional role of this putative F-specific protein and to elucidate the mechanisms of mt genome-specific mtDNA transmission in bivalve species with DUI.

### Conclusion

A fundamental question regarding doubly uniparental inheritance of mtDNA in bivalves is whether there are F- and/or M-specific mtDNA sequences that control the mode of inheritance of the mitochondrial genomes. Our results demonstrate that there is a systematic difference between maternally and paternally transmitted mytilid genomes: a fourteenth mtDNA-encoded protein, i.e., F-*ORF-VD1*, is likely present in the former but absent in the latter. Interestingly, this putative additional protein has been found in the first variable domain VD1 of the mitochondrial control region, which is the portion of the CR that was previously suspected to contain the elements responsible for the differing modes of mt transmission in DUI-containing bivalves [39], [40]. We present multiple lines of evidence suggesting that a functional protein is coded for by F-*ORF-VD1*: (i) the gene region has been maintained in the *Mytilus* lineage (subfamily Mytilinae) for at least 13 million years and our results suggest that a mytiline F-*ORF* homologue is present in *Musculista senhousia* (subfamily Crenellinae), (ii) the gene region has been classified as coding by testcode and Glimmer analyses, (iii) the gene region is actively transcribed in *Mytilus*, (iv) the putative protein's secondary structure has been conserved, (v) the putative protein's amino acid composition are relatively similar and (vi) the gene region's Ka/Ks ratios indicate relaxed purifying selection, which would not be expected for a non-protein coding sequence. Although it is admittedly speculative, we propose that F-*ORF-VD1* is essential for the maternal transmission of the F mitochondrial genome in mytilid mussels. Despite the fact that the function(s) of the F-*ORF-VD1* protein remains to be determined, our findings suggest that the functional repertoire of animal mitochondrial genomes is greater than previously thought and that novel mitochondrial ORFs, with key biological functions, await discovery in other animal groups.

## Materials and Methods

Complete mitochondrial genome sequences used in this study are listed in Table 1. Complete F- and M-type CR sequences of *M. californianus* (AF090831 [55]; AY515226-27 and EU826123-24 [40]) and the F-type CR sequence of *M. coruscus* (AF315574; Barna and Showman unpublished) have also been used. Because the complete *M. trossulus* F and M mt genomes sequenced by Zbawicka et al. [96] are introgressed mtDNAs from *M. edulis*, we used the genomes more recently sequenced by Zbawicka et al. [97] as the “ancestral *M. trossulus*” F and M mtDNAs.

Examination of ORFs was performed with ORF Finder (http://www.ncbi.nlm.nih.gov/projects/gorf/) using the invertebrate mitochondrial genetic code. Sequence similarity searches were performed in GenBank using BLASTX and PSI-BLAST [87] against the following databases on September 2010 (GenBank release 179.0): (i) non-redundant protein sequences, (ii) SWISSPROT (SWISSPROT release 2010_09), (iii) protein data bank and (iv) environmental samples. We also performed sequence similarity searches using BLASTN against expressed sequence tags (EST others) [98]. T-COFFEE version 8.93 [99] was used to align amino acid sequences and aa alignments were used as a template to align the corresponding codons. Graphical presentation of conserved positions in the alignment was done using Jalview [100].

The coding potential of ORFs was examined using Fickett's testcode algorithm [52] and Glimmer 3 [53]. Transmembrane helices as well as other ORF features of were characterized using HMMTOP [101], TMpred [102] and PredictProtein [103]. Hydropathy profiles were calculated using the method of Kyte and Doolittle [104]. Protein structure and function predictions were made using I-TASSER, a state-of the-art hierarchical protein structure modeling approach that is based on the secondary-structure enhanced profile-profile threading alignment [105], [106]. Amino acid composition for chemically equivalent amino acids was obtained following Taylor [107]: acidic amino acids (D and E); aromatic (H, F, W and Y); basic (R, H, and K); charged (R, D, E, H and K); hydrophilic (D, E, K, N, Q and R); hydrophobic (A, C, F, I, L, M, V, W and Y); neutral (G, Q, H, S and T); non-polar (A, C, G, I, L, M, F, P, V, W and Y); and polar (R, N, D, E, Q, H, K, S and T).

MEGA 4.0 [108] was used to estimate nucleotide and amino acid divergences among putative ORFs. The number of synonymous substitutions per synonymous site (Ks) and the number of nonsynonymous substitutions per nonsynonymous site (Ka) for the entire F-*orf-vd1* sequences within and between *Mytilus* spp. were also calculated using MEGA 4.0. Site-specific selection, i.e. the estimation of Ka/Ks ratios at each codon site, was studied with the SELECTON server 2.4 (http://selecton.tau.ac.il/index.html) using a Bayesian inference approach [81]. Specifically, the analysis was performed by means of a comparison between a null model assuming no positive selection (M8a; [109]) and a model that allows positive selection (MEC, which treats amino-acid replacements differently by allowing a position with radical replacements to obtain higher Ka value than a position with more moderate replacement; [110]). As data sources we used the codon-aligned partial *cox1* and complete F-*orf-vd1 Mytilus* spp. sequences (*M. senhousia* was excluded due to alignment issues) and the inferred ML phylogenetic tree. For our models (MEC vs. M8a), likelihood was tested by Akaike Information Criterion (AICc) score comparison [111]. The MEC model is considered the more justified if its AICc score is lower than the score of the alternative model. For each position, a confidence interval defined by the 5th and 95th percentiles of the posterior distributions inferred for the position was estimated. For positions with an inferred Ka/Ks>1, the inference of positive selection is considered reliable when the lower bound of the confidence interval is larger than 1.

## Supporting Information

Figure S1 **Examples of M-** ***ORF-VD1*** **sequences in GenBank.**
(DOC)

Figure S2 **Full length and Truncated F-** ***ORF-VD1*** **in GenBank.**
(DOC)

Figure S3 **Full alignment and Bayesian Ka/Ks ratios obtain from the MEC model – “SELECTON analysis”.**
(DOC)

Figure S4 **(A) Comparisons of** ***Mytilus edulis*** **F-** ***ORF-VD1*** **and** ***Venustaconcha ellipsiformis*** **F-** ***ORF*** **hydropathy profiles.** Profiles were calculated by the method of Kyte and Doolittle [104]. Numbers below profiles designate amino acid positions in each protein. Predicted transmembrane domains according to TMpred [102] are shown in light gray. (B) Alignment of the translated F-*orf-vd1 M. edulis* and F-*orf V. ellipsiformis* sequences. Identical amino acids are highlighted in black. Chemically equivalent amino acids are in gray. Dashes (–) denote a missing residue at this position in comparison with other sequence(s). (C) Overall amino acid composition (left) and composition of chemically equivalent amino acids (right) of *Mytilus* spp. F-*ORF-VD1*, *M. senhousia* F-*ORF* and *V. ellipsiformis* F-*ORF* protein sequences. Amino acid composition is reported as percentage.
(TIF)
