# Supplementary material for: Evidence for a Fourteenth mtDNA-Encoded Protein in the Female-Transmitted mtDNA of Marine Mussels (Bivalvia: Mytilidae)
Source: PLoS One. 2011 Apr 27;6(4):e19365. doi: 10.1371/journal.pone.0019365 (PMC3083442; doi:10.1371/journal.pone.0019365)
Supplement: Figure S1 — Examples of M- ORF-VD1 sequences in GenBank. (DOC) [file pone.0019365.s001.doc]

**Examples of *M. edulis* M-*ORF-VD1* sequences**

**AY823623**

MSSNSHLEKACFLGMWGVCSNNKLIPGESCKSFKNSNKATQKMWCVACVYKYTQKKKKKKKKYHKCLKMSCFYTLNFPATACNSSSRLCPPVFLYVKVYCWHMRELLYXNNL

**AY823624**

MSGLFKQQTNFSGESCKSFKNSXKATQKMWCVACVYKYTQKKKKKKKKYHKCLKMSCFYTLNFPATACNSSSRLCPPVFLYVKVYCWHMRELLY

**AY350791**

MVCSVCMQVYAKKKNKKKKNTMNA

**AY350791**

MSCFYTLNFPATACNSSSRLCPPVFLYVKVYCWHMRELLY

**AY188279**

MSGLFKQQTNSSGEGCKSFKNSNKATQKMWCVACVYKYTQKKKKKKKNTMNV

**DQ198245**

MSYFYTLNFPATTCNSSSRLYPPVFLYAKVYCWHMWELLYWNNL

**Examples of *M. galloprovincialis* M-*ORF-VD1* sequences**

**HM027630**

MYTSMRKKKKKKKKKTVKCLGMSCFHTLSFLAIACNSSNRLCSSVFLHVKVPCWHTWEPPY

**AY363687**

MYADSFTLKKSVVSRMWKAYLNNSVIPGESGASLVKNSNKAT

**AY363687**

MGMSCFYTLSLLAIACDSSNRLSSPVFLHVKVPCWRTWEPPY

**Examples of *M. trossulus* M-*ORF-VD1* sequences**

**GQ438250**

MTCLDKKTSVSKPWGVFSVCACNKQKKKKKKKKKKKKPWKHLSMSLYGVLHFLATGSDSDHDPLPPVFLDAKPCCWCM

**DQ013366**

MSKQVYLNHGVCLGCVPVTNEKKKKKKKKKKEKTVKTFKNEALWRTSFPCHS

**DQ013366**

MSLYGVLHFLVTGSDNGHDPFPPVFLDAKPCCWCM

**AY515231**

MTCLDKKTSVSKPWGVFSVCACNKQKKQKKKKKKKKNRENP

**AF188282**

MTCLDKKTSVYKPWGVFSVCACNNAKKKKKKKKKKKKPWKHLSMSLYGVLHFLATGSDSDHGPFPPVFLDAKPCCWCM

**EU826077**

MTCLDKKTSVSKPWGVFSVCACNKQKKKKKKKKSKNRENP

**Examples of *M. californianus* M-*ORF-VD1* sequences**

**AF188284**

MNFMSCKKRLYKYTNVCTLSDCLKKYLSSVPL

**AF188284**

MLMCVLWVTALKSIYLPCHYKCSYKMQQSM

***M. coruscus* M-*ORF-VD1* sequences**

**AF315572**

MSHSCLRLDQSKSMAISWVQSHVSLSYFLGMKLMILILIF
