# Supplementary material for: Evidence for a Fourteenth mtDNA-Encoded Protein in the Female-Transmitted mtDNA of Marine Mussels (Bivalvia: Mytilidae)
Source: PLoS One. 2011 Apr 27;6(4):e19365. doi: 10.1371/journal.pone.0019365 (PMC3083442; doi:10.1371/journal.pone.0019365)
Supplement: Figure S2 — Full length and Truncated F- ORF-VD1 in GenBank. (DOC) [file pone.0019365.s002.doc]

**Full length *M. californianus* F-*ORF-VD1* (129aa, n = 3)**

*M. californianus* **AY515227 – AF188283 – EU826123**

MTIFIIEMTLLLWNFDMIEHFLMFCKSFLESEEWMLSLPHDGFSRVIPSFSMDSGGSGDLYSGGGGDSVEVASSSEPVSAGGEGPVSGVTEVTPNTMSSQEVGIVEGPMSVVQSESSNPEASCENKESS

**Full length *M. trossulus* F-*ORF-VD1* (153aa, n = 12)**

*M. trossulus* ***Accession numbers are indicated below**

MSVLLSDSLLNVLGTSEAVWEWLSQGFAAKKGLLLSGVWDGFFSYKNWVFSMDVGGGDLCQGGGGDTVSVLPLPETISAAGDAVVNGVAEVVPDNQEEGGPHAEGGYVPLEEQVAVVEPEVLANVCQPVEQGNVVVSEEDSVPDVSKDGVSSY

**Truncated *M. trossulus* F-*ORF-VD1* (109aa, n = 3)**

*M. trossulus* **AY515230 – EU826073 – EU826074**

MSVLLSDSLLNVLGTSEAVWEWLSQGFAAKKGLLLSGVWDGFFSYKNWVFSMDVGGGDLCQGGGGDTVSVLPLPETISAAGDAVVNGVAEVVPDNQEEG*APMLSVAMFL*


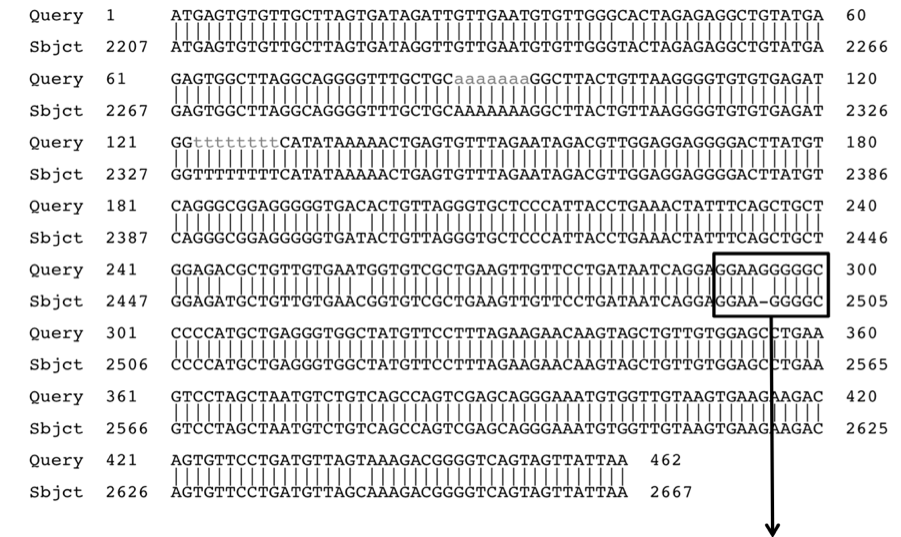


*Deletion at position 295 in truncated M. trossulus F-orf-vd1 has been found in 3 out of 15 complete M. trossulus vd1 sequences available in GenBank. The same deletion has not been found in 156 partially sequenced M. trossulus vd1 sequences available in GenBank, which correspond to the first 407nt of the* F-*orf-vd1 and, when translated, to the first 135aa of the F-ORF-VD1 without any stop codon.* ***Accession numbers for partially sequenced *M. trossulus vd1* sequences are indicated below.**

**Full length *M. edulis* or *M. galloprovincialis* F-*ORF-VD1* (163aa, n = 41)**

*M. edulis* ***Accession numbers are indicated below**

MSMLFGDSLLSVVDFSEVLCSWFKAGFLVKKDLLLSGVWDTFLSHKNSMFGMDAGDGGLCQGGEGDGAQVRVTPEAVWVGGDTAVNAGAEAAPDNAEGAGRYVGDGYALPLEEVGCSSVEESESAVAEPEVVSSGFEPVEQSGVLISEASGAINAGKESFSDC

**Truncated *M. edulis* F-*ORF-VD1* (84aa to 144aa found in 7 recombinant CR only in males mussels)**

*M. edulis* **DQ198226**

MSMLFGDSLLSVVDFSEVLCSWFKAGFLVKKDLLLSGVWDTFLSHKNSMFGMDAGDGGLCQGGEGDGAQVRVTPEAVWGGGDTPVNAGAEA*LLMMRSSRGGTLETAMPFR*

*M. edulis* **DQ198244**

MNMLFSDSLLSVVDFSGVLCSWFKAGFLVKKDLLLSSVWDTFLSHKNSMFGMDAGDGGLCQGSEGDGAQARVIPEAVWGGGDSAVNVGAEAAPDNAEGAGWNVGDGYALPVEEVGCSSVEESESAVVEPEVVSSG*LSRSSSEVC*

*M. edulis* **DQ198245**

MNMLFSDSLLSVVDFKGVLCSWFKAGFLVKKDLLLSSVWDIFLSHKNSMFGMDAGDGGLCQGGEGD*VPKHVLFLKLYGVVETQL*

*M. edulis* **DQ198247**

MNMLFSDSLLSVVDFSGVLCSWFKAGFLVKKDLLLSSVWDTFLSHKNSMFGMDAGDGGLCQGGEGDGAQARVIPEAVWGGGDTAVNAGAEAAPDNAEG*RGGTLETAMPFR*

*M. edulis* **DQ198248**

*MSS*VWDTFLSHKNSMFGMDAGDGGLCQGGEGDGAQARVIPEAVWGGGDTAVNAGAEAAPDNAEGAGWNAGDGYALPVEEVGCSSVEESESAVVEPEVVSSGFEPVEQSGVLISEASGAINAGKESFSDC

*M. edulis* **DQ198249**

*MSS*VWDTFLSHKNSMFGMDAGDGGLCQGGEGDGAQARVIPEAVWGGGDTAVNAGAEAAPDNAEGAGWNAGDGYALPVEEVGCSSVEESESAVVEPEVVSSGFEPVEQSGVLISEASGAINAGKESFSDC

*M. edulis* **DQ198251**

MNMLFSDSLLSVVDFSGVLCSWFKAGFLVKKDLLLSSVWDTFLSHKNSMFGMDAGDG*AYVSVEKVTVPKHVLFLKLYGVVETQL*

**Truncated *M. edulis* F-*ORF-VD1* found in one non-recombinant CR in a female**

*M. edulis* **AY484747**

--------MLSVVDFSEVLCSWFKAGFLVKKDLLLSGVWDTFLSHKNSMFGMDAGDGGLCQGGEGDG

AQARVTPEAVWGGGDTPVNAGAEAAPDNAEEAGGYVGD*AMPFR*

***Accession numbers for full length *M. trossulus* F-*ORF-VD1*:**

GU936625

EU826072-EU826075-EU826076

AY823655

AY636148, AY636150 to AY636153

HM462080

AF188281

***Accession numbers for partially sequenced *M. trossulus* F-*ORF-VD1*:**

DQ379517 to DQ379672

***Accession numbers for full length *M. edulis/M. galloprovincialis* F-*ORF-VD1* (some sequences are identified as *M. trossulus* but derive through recent introgression from *M. edulis*):**

AY629165

AY115479

AY497292

AF315573

AY350784 to AY350788, AY350790

DQ403168

DQ198227 to DQ198230, DQ198232 to DQ198235, DQ198246, DQ198250, DQ198252 to DQ198255, DQ198257, DQ198258

EF434630, EF434638 to EF434649, EF434653
