# Supplementary material for: Evidence for a Fourteenth mtDNA-Encoded Protein in the Female-Transmitted mtDNA of Marine Mussels (Bivalvia: Mytilidae)
Source: PLoS One. 2011 Apr 27;6(4):e19365. doi: 10.1371/journal.pone.0019365 (PMC3083442; doi:10.1371/journal.pone.0019365)
Supplement: Figure S3 — Full alignment and Bayesian Ka/Ks ratios obtain from the MEC model – “SELECTON analysis”. (DOC) [file pone.0019365.s003.doc]

**DNA alignment used for the “SELECTON analysis”**

>DL; 'Mytcorcox1/1-893'

'Mytcorcox1/1-893'

CTGATAATTCGGATGCAGTTAGGGCACCCTGGAGCAGTTTTTTTAAAAAGGGACTGATTTTATAATGTAGTAGTA

ACAACGCATGCTCTGATAATAATTTTCTTTGCTGTGATACCCATTTTAATTGGGGCCTTCGGTAACTGGCTTATT

CCTTTGCTTGTTGGGGGAAAAGACATAATCTATCCACGTATAAACAATCTTAGATATTGACTGTCGCCGAATGCT

CTATATTTATTGATGTTATCTTTTAGGACAGATAAAGGAGTGGGGGCAGGGTGGACGATTTATCCTCCTCTATCT

GTGTACCCCTATCATAGAGGGCCCAGAATGGATGTTCTTATTGTGTCTCTTCATCTGGCAGGACTCAGGTCTTTA

GTGGGAGCTATTAATTTTGCTAGCACAAATAAGAACATACCAGTATTAGAAATAAAAGGGGAACGGGCTGAGCTT

TATGTGCTAAGAATCAGAGTTACTGCGGTTCTTTTAATTATTTCGATTCCGGTGTTAATG---------------

---------TTGTGCCTATGAAATTTTAGTGTTGTTGAACATCTTCTGATTTTTAGTGAATTGACTCTAGGTACT

GGGGTTTGTTTGTTAAACTTGACTTATGAGGGGTTT---TCTTTAATAAACCTGAGCTTTTTGATAGATTCAGGA

GGA---GGAGATTTGTGCTCAGCGCGAGGGGGTGACAGGAGCGACGTAGTTAGGAGTCCTCAGCCTGTGTCAGCG

AGGGGGGAGAGGTCTATGAGAGGAGAGGCTGAAGTTGCTCCTAATAGACTTAGACCA------------------

---------------------ACG------------------------------GAGGTTGAATCA---------

---------------------------GTTGAAGCGTCAGAATCTGTGACGCAGAGGGAAAGGATTAATCAAGAA

ATTTCCTGTGAAACAAGAGAAAAGACACAAGAACAGCTCTTTTTGGGCTAA*

>DL; 'Mytcal3/1-899'

'Mytcal3/1-899'

TTGATAATTCGAATGCAATTAGGGCATCCTGGAGCAGTCTTCCTAAAAAGAGATTGATTTTATAATGTGGTTGTT

ACAACACATGCTTTAATAATAATTTTCTTTGCTGTAATACCCATTTTAATCGGGGCTTTTGGTAATTGGCTTATT

CCTTTGCTTGTAGGGGGAAAAGACATAATCTATCCACGTATAAATAATTTGAGATATTGACTGTCGCCAAATGCA

CTATATTTATTAATGCTATCTTTTAGAACAGATAAAGGAGTAGGGGCAGGATGGACTATTTATCCACCTCTATCT

GTATACCCTTATCACAGAGGGCCTAGGATAGATGTTCTTATTGTGTCTTTACACTTAGCAGGACTTAGCTCTCTA

GTGGGGGCAATTAATTTTGCTAGCACAAATAAAAATATACCAGTATTAGAAATAAAAGGGGAACGGGCGGAGCTC

TATGTGTTAAGAATTAGGGTTACTGCAGTTCTTTTAATTATCTCAATTCCAGTGTTAATGACTATTTTCATTATC

GAAATAACTCTGCTTCTATGAAACTTTGATATAATTGAGCATTTTTTAATATTTTGTAAATCGTTTTTAGAATCT

GAGGAGTGAATGTTAAGCCTGCCTCATGATGGGTTT---TCACGAGTAATTCCAAGTTTTTCTATGGACTCAGGA

GGAAGGGGAGATCTTTATTCAGGGGGAGGAGGCGACAGTGTGGAAGTAGCTAGAAGTTCCGAGCCTGTGTCAGCT

GGAGGGGAGGGGCCCGTGAGAGGGGTAACTGAAGTTACTCCTAATACCATGAGATCC------------------

---------------------CAA------------------------------GAAGTTGGGATC---------

---------------------------GTTGAAGGGCCTATGTCTGTGGTGCAGAGAGAAAGGTCGAACCCTGAA

GCTTCTTGTGAAAATAAAGAAAGAAGC---------------------TAG*

>DL; 'Mytcal1/1-899'

'Mytcal1/1-899'

TTGATAATTCGAATGCAATTAGGGCATCCTGGAGCAGTCTTCCTAAAAAGAGATTGATTCTATAATGTGGTTGTT

ACAACACATGCTTTAATAATAATTTTCTTTGCTGTAATACCAATTTTAATCGGGGCTTTTGGTAATTGGCTTATT

CCTTTGCTTGTAGGGGGAAAAGACATAATCTATCCACGTATAAATAATTTGAGATATTGACTGTCGCCAAATGCA

CTATATTTATTAATGCTATCTTTTAGAACAGATAAAGGAGTAGGGGCAGGATGGACTATTTATCCACCTCTATCT

GTATACCCTTATCACAGAGGGCCTAGGATAGATGTTCTTATTGTGTCTTTACACTTAGCAGGACTTAGCTCTCTA

GTGGGGGCAATTAATTTTGCTAGCACAAATAAAAATATACCAGTATTAGAAATAAAAGGGGAACGGGCGGAGCTC

TATGTGTTAAGAATTAGGGTTACTGCAGTTCTTTTAATTATCTCAATTCCAGTGTTAATGACTATTTTCATTATC

GAAATAACTCTGCTTCTATGAAACTTTGATATAATTGAGCATTTTTTAATATTTTGTAAATCGTTTTTAGAATCT

GAGGAGTGAATGTTAAGCCTGCCTCATGATGGGTTT---TCACGAGTAATTCTAAGTTTTTCTATGGACTCAGGA

GGAAGGGGAGATCTTTATTCAGGGGGAGGAGGCGACAGTGTGGAAGTAGCTAGAAGTTCCGAGCCTGTGTCAGCT

GGAGGGGAGAGGCCCGTGAGAGGGGTAACTGAAGTTACTCCTAATACCATGAGATCC------------------

---------------------CAA------------------------------GAAGTTGGGATC---------

---------------------------GTTGAAGGGCCTGTGTCTGTGGTGCAGAGAGAAAGGTCGAACCCTGAA

GCTTCTTGTGAAAATAAAGAAAGAAGC---------------------TAG*

>DL; 'Mytcal2/1-899'

'Mytcal2/1-899'

TTGATAATTCGAATGCAATTAGGGCATCCTGGAGCAGTCTTCCTAAAAAGAGATTGATTCTATAATGTGGTTGTT

ACAACACATGCTTTAATAATAATTTTCTTTGCTGTAATACCAATTTTGATCGGGGCTTTTGGTAATTGGCTTATT

CCTTTGCTTGTAGGGGGAAAAGACATAATCTATCCACGTATAAATAATTTGAGATATTGACTGTCGCCAAATGCA

CTATATTTATTAATGCTATCTTTTAGAACAGATAAAGGAGTAGGGGCAGGATGGACTATTTATCCCCCTCTATCT

GTATACCCTTATCACAGAGGGCCTAGGATAGATGTTCTTATTGTGTCTTTACACTTAGCAGGACTTAGCTCTCTA

GTGGGGGCAATTAATTTTGCTAGCACAAATAAAAATATACCAGTATTAGAAATAAAAGGGGAACGGGCGGAGCTC

TATGTGTTAAGAATTAGGGTTACTGCAGTTCTTTTAATTATCTCAATTCCAGTGTTAATGACTATTTTCATTATC

GAAATAACTCTGCTTCTATGAAACTTTGATATAATTGAGCATTTTTTAATATTTTGTAAATCGTTTTTAGAATCT

GAGGAGTGAATGTTAAGCCTGCCTCATGATGGGTTT---TCACGAGTAATTCTAAGTTTTTCTATGGACTCAGGA

GGAAGGGGAGATCTTTATTCAGGGGGAGGAGGCGACAGTGTGGAAGTAGCTAGAAGTTCCGAGCCTGTGTCAGCT

GGAGGGGAGGGGCCCGTGAGAGGGGTAACTGAAGTTACTCCTAATACCATGAGATCC------------------

---------------------CAA------------------------------GAAGTTGGGATC---------

---------------------------GTTGAAGGGCCTGTGTCTGTGGTGCAGAGAGAAAGGTCGAACCCTGAA

GCTTCTTGTGAAAATAAAGAAAGAAGC---------------------TAG*

>DL; 'Mtros3/1-971'

'Mtros3/1-971'

TTAATGATCCGAATGCAACTGGGTCATCCTGGAGCAGTGTTCTTAAAAAGAGATTGATTCTATAATGTGGTGGTT

ACAACGCATGCCTTAATAATAATTTTTTTTGCTGTGATACCTATCTTAATTGGAGCTTTCGGTAATTGGTTGATT

CCTCTGCTAGTAGGAGGTAAAGATATAATTTACCCGCGAATAAATAACTTAAGCTATTGACTATCTCCTAATGCA

CTATATTTACTAATACTGTCCTTTAGAACGGACAAAGGAGTTGGTGCTGGATGAACTATTTACCCCCCTTTATCT

GTGTACCCCTATCATAGGGGCCCTAGGATAGATGTTCTTATTGTGTCACTACATCTAGCTGGGCTCAGCTCTCTA

GTGGGGGCTATTAACTTTGCTAGGACCAATAAAAATATGCCAGTGTTAGAAATGAAAGGAGAACGAGCGGAGCTT

TATGTTTTAAGGATTAGAGTTACTGCAGTTCTTTTAATTATTTCAATTCCGGTTCTAATGAGTGTGTTGCTTAGT

GATAGATTGTTGAATGTGTTGGGCACTAGAGAGGCTGTATGAGAGTGG---CTTAGGCAGGGGTTTGCTGCAAAG

AAAGGCTTACTGTTAAGGGGTGTGTGAGATGGTTTTTTTCCATATAAAAACTGAGTGTTTAGAATAGACGTTGGA

GGA---GGGGACTTATGTCAGGGCGGAGGGGGTGACACTGTTAGGGTGCTCCCATTACCTGAAACTATTTCAGCT

GCTGGAGATGCTGTTGTGAATGGTGTCGCTGAAGTTGTTCCTGATAATCAGGAGGAAGGGGGCCCCCATGCTGAG

GGTGGCTAT---GTTCCTTTAGAAGAA---------------------------CAAGTAGCTGTTGTGGAGCCT

GAAGTCCTAGCTAATGTCTGTCAGCCAGTCGAGCAGAGAAATGTGGTTGTAAGTGAAGAAGACAGTGTTCCTGAT

GCTAGTAAAGACGGGGTCAGTAGTTAT---------------------TAA*

>DL; 'Mtros6/1-971'

'Mtros6/1-971'

TTAATGATCCGAATGCAACTGGGTCATCCTGGAGCAGTGTTCTTAAAAAGAGATTGATTCTATAATGTGGTGGTT

ACAACGCATGCCTTAATAATAATTTTTTTTGCTGTGATACCTATCTTAATTGGAGCTTTCGGTAATTGGTTGATT

CCTCTGCTAGTAGGAGGTAAAGATATAATTTACCCGCGAATAAATAACTTAAGTTATTGACTATCTCCTAATGCA

CTATATTTGCTAATACTGTCCTTTAGAACGGATAAAGGAGTTGGTGCTGGATGAACTATTTACCCCCCTTTATCT

GTGTACCCCTATCATAGGGGCCCTAGGATAGATGTTCTTATTGTGTCACTACATCTAGCTGGGCTCAGCTCTCTA

GTGGGGGCTATTAACTTTGCTAGGACCAATAAAAATATGCCAGTGTTAGAAATGAAAGGAGAACGAGCGGAGCTT

TATGTTTTAAGGATTAGAGTTACTGCAGTTCTTTTAATTATTTCAATTCCGGTTTTAATGAGTGTGTTGCTTAGT

GATAGATTGTTGAATGTGTTGGGCACTAGAGAGGCTGTATGAGAGTGG---CTTAGGGAGGGGTTTGCTGCAAAA

AAAGGCTTACTGTTAAGGGGTGTGTGAGATGGTTTTTTTTCATATAAAAACTGAGTGTTTAGAATAGACGTTGGA

GGA---GGGGACTTATGTCAGGGCGGAGGGGGTGACACTGTTAGGGTGCTCCCATTACCTGAAACTATTTCAGCT

GCTGGAGATGCTGTTGTGAATGGTGTCGCTGAAGTTGTTCCTGATAATCAGGAGGAAGGGGGCCCCCATGCTGAG

GGTGGCTAT---GTTCCTTTAGAAGAA---------------------------CAAGTAGCTGTTGTGGAGCCT

GAAGTCCTAGCTAATGTCTGTCAGCCAGTCGAGCAGGGAAATGTGGTTGTAAGTGAAGAAGACAGTGTTCCTGAT

GTTAGTAAAGACGGGGTCAGTAGTTAT---------------------TAA*

>DL; 'Mtros7/1-971'

'Mtros7/1-971'

TTAATGATCCGAATGCAACTGGGTCATCCTGGAGCAGTGTTCTTAAAAAGAGATTGATTCTATAATGTGGTGGTT

ACAACGCATGCCTTAATAATAATTTTTTTTGCTGTGATACCTATCTTAATTGGAGCTTTCGGTAATTGGTTGATT

CCTCTGCTAGTAGGAGGTAAAGATATAATTTACCCGCGAATAAATAACTTAAGTTATTGACTATCTCCTAATGCA

CTATATTTGCTAATACTGTCCTTTAGAACGGATAAAGGAGTTGGTGCTGGATGAACTATTTACCCCCCTTTATCT

GTGTACCCCTATCATAGGGGCCCTAGGATAGATGTTCTTATTGTGTCACTACATCTAGCTGGGCTCAGCTCTCTA

GTGGGGGCTATTAACTTTGCTAGGACCAATAAAAATATGCCAGTGTTAGAAATGAAAGGAGAACGAGCGGAGCTT

TATGTTTTAAGGATTAGAGTTACTGCAGTTCTTTTAATTATTTCAATTCCGGTTTTAATGAGTGTGTTGCTTAGT

GATAGATTGTTGAATGTGTTGGGCACTAGAGAGGCTGTATGAGAGTGG---CTTAGGCAGGGGTTTGCTGCAAAA

AAAGGCTTACTGTTAAGGGGTGTGTGAGATGGTTTTTTTTCATATAAAAACTGAGTGTTTAGAATAGACGTTGGA

GGA---GGGGACTTATGTCAGGGCGGAGGGGGTGACACTGTTAGGGTGCTCCCATTACCTGAAACTATTTCAGCT

GCTGGAGATGCTGTTGTGAATGGTGTCGCTGAAGTTGTTCCTGATAATCAGGAGGAAGGGGGCCCCCATGCTGAG

GGTGGCTAT---GTTCCTTTAGAAGAA---------------------------CAAGTAGCTGTTGTGGAGCCT

GAAGTCCTAGCTARTGTCTGTCAGCCAGTCGAGCAGgGAAATGTGGTTGTAAGTGAAGAAGACAGTGTTCCTGAT

GTTAGTAAAGACGGGGTCAGTAGTTAT---------------------TAA*

>DL; 'Mtros1/1-971'

'Mtros1/1-971'

TTAATGATCCGAATGCAACTGGGTCATCCTGGAGCAGTGTTCTTAAAAAGAGATTGATTCTATAATGTGGTGGTT

ACAACGCATGCCTTAATAATAATTTTTTTTGCTGTGATACCTATCTTAATTGGAGCTTTCGGTAATTGGTTGATT

CCTCTGCTAGTAGGAGGTAAAGATATAATTTACCCGCGAATAAATAACTTAAGTTATTGACTATCTCCTAATGCA

CTATATTTACTAATACTGTCCTTTAGAACGGATAAAGGAGTTGGTGCTGGATGAACTATTTACCCCCCTTTATCT

GTGTACCCCTATCATAGGGGCCCTAGGATAGATGTTCTTATTGTGTCACTACATCTAGCTGGGCTTAGCTCTCTA

GTGGGGGCTATTAACTTTGCTAGGACCAATAAAAATATGCCAGTGTTAGAAATGAAAGGAGAACGAGCGGAGCTT

TATGTTTTAAGGATTAGAGTTACTGCAGTTCTTTTAATTATTTCAATTCCGGTTCTAATGAGTGTGTTGCTTAGT

GATAGATTGTTGAATGTGTTGGGCACTAGAGAGGCTGTATGAGAGTGG---CTTAGGCAGGGGTTTGCTGCAAAA

AAAGGCTTACTGTTAAGGGGTGTGTGAGATGGTTTTTTTTCATATAAAAACTGAGTGTTTAGAATAGACGTTGGA

GGA---GGGGACTTATGTCAGGGCGGAGGGGGTGACACTGTTAGGGTGCTCCCATTACCTGAAACTATTTCAGCT

GCTGGAGACGCTGTTGTGAATGGTGTCGCTGAAGTTGTTCCTGATAATCAGGAGGAAGGGGGCCCCCATGCTGAG

GGTGGCTAT---GTTCCTTTAGAAGAA---------------------------CAAGTAGCTGTTGTGGAGCCT

GAAGTCCTAGCTAATGTCTGTCAGCCAGTCGAGCAGGGAAATGTGGTTGTAAGTGAAGAAGACAGTGTTCCTGAT

GTTAGTAAAGACGGGGTCAGTAGTTAT---------------------TAA*

>DL; 'Mtros2/1-971'

'Mtros2/1-971'

TTAATGATCCGAATGCAACTGGGTCATCCTGGAGCAGTGTTCTTAAAAAGAGATTGATTCTATAATGTGGTGGTT

ACAACGCATGCCTTAATAATAATTTTTTTTGCTGTGATACCTATCTTAATTGGAGCTTTCGGTAATTGGTTGATT

CCTCTGCTAGTAGGAGGTAAAGATATAATTTACCCGCGAATAAATAACTTAAGTTATTGACTATCTCCTAATGCA

CTATATTTACTAATACTGTCCTTTAGAACGGATAAAGGAGTTGGTGCTGGATGAACTATTTACCCCCCTTTATCT

GTGTACCCCTATCATAGGGGCCCTAGGATAGATGTTCTTATTGTGTCACTACATCTAGCTGGGCTCAGCTCTCTA

GTGGGGGCTATTAACTTTGCTAGGACCAATAAAAATATGCCAGTGTTAGAAATGAAAGGAGAACGAGCGGAGCTT

TATGTTTTAAGGATTAGAGTTACTGCAGTTCTTTTAATTATTTCAATTCCGGTTCTAATGAGTGTGTTGCTTAGT

GATAGATTGTTGAATGTGTTGGGCACTAGAGAGGCTGTATGAGAGTGG---CTTAGGCAGGGGTTTGCTGCAAAA

AAAGGCTTACTGTTAAGGGGTGTGTGAGATGGTTTTTTTTCATATAAAAACTGAGTGTTTAGAATAGACGTTGGA

GGA---GGGGACTTATGTCAGGGCGGAGGGGGTGACACTGTTAGGGTGCTCCCATTACCTGAAACTATTTCAGCT

GCTGGAGATGCTGTTGTGAATGGTGTCGCTGAAGTTGTTCCTGATAATCAGGAGGAAGGGGGCCCCCATGCTGAG

GGTGGCTAT---GTTCCTTTAGAAGAA---------------------------CAAGTAGCTGTTGTGGAGCCT

GAAGTCCTAGCTAATGTCTGTCAGCCAGTCGAGCAGGGAAATGTGGTTGTAAGTGAAGAAGACAGTGTTCCTGAT

GTTAGTAAAGACGGGGTCAGTAGTTAT---------------------TAA*

>DL; 'Mtros3/1-971'

'Mtros3/1-971'

TTAATGATCCGAATGCAACTGGGTCATCCTGGAGCAGTGTTCTTAAAAAGAGATTGATTCTATAATGTGGTGGTT

ACAACGCATGCCTTAATAATAATTTTTTTTGCTGTGATACCTATCTTAATTGGAGCTTTCGGTAATTGGTTGATT

CCTCTGCTAGTAGGAGGTAAAGATATAATTTACCCGCGAATAAATAACTTAAGTTATTGACTATCTCCTAATGCA

CTATATTTACTAATACTGTCCTTTAGAACGGATAAAGGAGTTGGTGCTGGATGAACTATTTACCCCCCTTTATCT

GTGTACCCCTATCATAGGGGCCCTAGGATAGATGTTCTTATTGTGTCACTACATCTAGCTGGGCTCAGCTCTCTA

GTGGGGGCTATTAACTTTGCTAGGACCAATAAAAATATGCCAGTGTTAGAAATGAAAGGAGAACGAGCGGAGCTT

TATGTTTTAAGGATTAGAGTTACTGCAGTTCTTTTAATTATTTCAATTCCGGTTCTAATGAGTGTGTTGCTTAGT

GATAGATTGTTGAATGTGTTGGGCACTAGAGAGGCTGTATGAGAGTGG---CTTAGGCAGGGGTTTGCTGCAAAA

AAAGGCTTACTGTTAAGGGGTGTGTGAGATGGTTTTTTTTCATATAAAAACTGAGTGTTTAGAATAGACGTTGGA

GGA---GGGGACTTATGTCAGGGCGGAGGGGGTGACACTGTTAGGGTGCTCCCATTACCTGAAACTATTTCAGCT

GCTGGAGATGCTGTTGTGAATGGTGTCGCTGAAGTTGTTCCTGATAATCAGGAGGAAGGGGGCCCCCATGCTGAG

GGTGGCTAT---GTTCCTTTAGAAGAA---------------------------CAAGTAGCTGTTGTGGAGCCT

GAAGTCCTAGCTAATGTCTGTCAGCCAGTCGAGCAGGGAAATGTGGTTGTAAGTGAAGAAGACAGTGTTCCTGAT

GTTAGTAAAGACGGGGTCAGTAGTTAT---------------------TAA*

>DL; 'Mtros4/1-971'

'Mtros4/1-971'

TTAATGATCCGAATGCAACTGGGTCATCCTGGAGCAGTGTTCTTAAAAAGAGATTGATTCTATAATGTGGTGGTT

ACAACGCATGCCTTAATAATAATTTTTTTTGCTGTGATACCTATCTTAATTGGAGCTTTCGGTAATTGGTTGATT

CCTCTGCTAGTAGGAGGTAAAGATATAATTTACCCGCGAATAAATAACTTAAGTTATTGACTATCTCCTAATGCA

CTATACTTACTAATACTGTCCTTTAGAACGGATAAAGGAGTTGGTGCTGGATGAACTATTTACCCCCCTTTATCT

GTGTACCCCTATCATAGGGGCCCTAGGATAGATGTTCTTATTGTGTCACTACATCTAGCTGGGCTTAGCTCTCTA

GTGGGGGCTATTAACTTTGCTAGGACCAATAAAAATATGCCAGTGTTAGAAATGAAAGGAGAACGAGCGGAGCTT

TATGTTTTAAGGATTAGAGTTACTGCAGTTCTTTTAATTATTTCAATTCCGGTTTTAATGAGTGTGTTGCTTAGT

GATAGATTGTTGAATGTGTTGGGCACTAGAGAGGCTGTATGAGAGTGG---CTTAGGCAGGGGTTTGCTGCAAAA

AAAGGCTTACTGTTAAGGGGTGTGTGAGATGGTTTTTTTTCATATAAAAACTGAGTGTTTAGAATAGACGTTGGA

GGA---GGGGACTTATGTCAGGGCGGAGGGGGTGACACTGTTAGGGTGCTCCCATTACCTGAAACTATTTCAGCT

GCTGGAGATGCTGTTGTGAATGGTGTCGCTGAAGTTGTTCCTGATAATCAGGAGGAAGGGGGCCCCCATGCTGAG

GGTGGCTAT---GTTCCTTTAGAAGAA---------------------------CAAGTAGCTGTTGTGGAGCCT

GAAGTCCTAGCTAATGTCTGTCAGCCAGTCGAGCAGGGAAATGTGGTTGTAAGTGAAGAAGACAGTGTTCCTGAT

GTTAGTAAAGACGGGGTCAGTAGTTAT---------------------TAA*

>DL; 'Mtros5/1-971'

'Mtros5/1-971'

TTAATGATCCGAATGCAACTGGGTCATCCTGGAGCAGTGTTCTTAAAAAGAGATTGATTCTATAATGTGGTGGTT

ACAACGCATGCCTTAATAATAATTTTTTTTGCTGTGATACCTATCTTAATTGGAGCTTTCGGTAATTGGTTGATT

CCTCTGCTAGTAGGAGGTAAAGATATGATTTACCCGCGAATAAATAACTTAAGTTATTGACTATCTCCTAATGCA

CTATACTTACTAATACTGTCCTTTAGAACGGATAAAGGAGTTGGTGCTGGATGAACTATTTACCCCCCTTTATCT

GTGTACCCCTATCATAGGGGCCCTAGGATAGATGTTCTTATTGTGTCACTACATCTAGCTGGGCTCAGCTCTCTA

GTGGGGGCTATTAACTTTGCTAGGACCAATAAAAATATGCCAGTGTTAGAAATGAAAGGAGAACGAGCGGAGCTT

TATGTTTTAAGGATTAGAGTTACTGCAGTTCTTTTAATTATTTCAATTCCGGTTTTAATGAGTGTGTTGCTTAGT

GATAGATTGTTGAATGTGTTGGGCACTAGAGAGGCTGTATGAGAGTGG---CTTAGGCAGGGGTTTGCTGCAAAA

AAAGGCTTACTGTTAAGGGGTGTGTGAGATGGTTTTTTTTCATATAAAAACTGAGTGTTTAGAATAGACGTTGGA

GGA---GGGGACTTATGTCAGGGCGGAGGGGGTGACACTGTTAGGGTGCTCCCATTACCTGAAACTATTTCAGCT

GCTGGAGATGCTGTTGTGAATGGTGTCGCTGAAGTTGTTCCTGATAATCAGGAGGAAGGGGGCCCCCATGCTGAG

GGTGGCTAT---GTTCCTTTAGAAGAA---------------------------CAAGTAGCTGTTGTGGAGCCT

GAAGTCCTAGCTAATGTCTGTCAGCCAGTCGAGCAGGGAAATGTGGTTGTAAGTGAAGAAGACAGTGTTCCTGAT

GTTAGTAAAGACGGGGTCAGTAGTTAT---------------------TAA*

>DL; 'Medu6/1-1001'

'Medu6/1-1001'

TTGATAATCCGCATACAACTTGGTCACCCTGGAGCGGTCTTTCTCAAAAGAGACTGGTTTTTTAATGTAGTGGTT

ACAACACATGCCTTAATGATGATTTTTTTTGCCGTAATACCAATCTTAATCGGGGCTTTTGGCAATTGGCTTATC

CCGTTGTTGGTAGGAGGTAAGGATATAATTTATCCCCGGATGAACAATTTGAGATATTGATTGTCTCCAAACGCA

TTGTACTTACTCATATTATCTTTTAGGACAGATAAAGGGGTAGGTGCTGGATGAACTGTCTACCCCCCACTATCC

AGGTACCCGTACCATAGAGGGCCAAGAATGGACGTTCTTATTGTGGCTCTTCATTTAGCTGGAGTAAGGTCTCTA

GTAGGGGCTATTAATTTTGCTAGTACTAACAAAAACATACCGGCCTTGGAGATAAAAGGGGAGCGAGCCGAGCTC

TATGTCTTAAGAATCAGGATCACTGCAGCCCTTCTAATCATTTCTATTCCAGTTCTAATGAGCATATTGTTTGGT

GATAGGTTGTTAAGTGTGGTAGATTTTAGAGAAGTCCTATGTAGCTGG---TTTAAAGCAGGCTTTTTAGTAAAA

AAAGACCTGCTGTTGAGGAGAGCGTGAGATACTTTTTTTTCACATAAAAATTCCATGTTTGGAATAGATGCAGGA

GAT---GGGGGCTTATGCCAGGGTGGAGAAGGTGACGGTGCCCAAGTACGTGTTACTCCTGAAGCTGTATGGGGT

GGTGGAGACACAGCTGTAAATGCGGGCGCCGAAGCCGCTCCTGATAATGCGGAGGAGGCGGGGTGGAACGTTGGA

GACGGCTATGCCCTTCCGTTAGAAGAAGTAGGTTGTAGTTCAGTTGAAGAAAGAGAAAGGGCTGTCGCGGAGCCT

GAAGTTGTATCTAGCGGTTCCGAGCCGGTCGAGCAGAGAGGTGTGCTAATTAGCGAAGCTAGTGGTGCCATTAAT

GCTGGCAAAGAAAGGTTTAGTGATTGT---------------------TAA*

>DL; 'Mgal1/1-1001'

'Mgal1/1-1001'

CTGATAATTCGGATACAGCTAGGGCATCCTGGAGCAGTATTTTTAAAAAGAGATTGGTTTTATAATGTGGTTGTT

ACAACACACGCCTTAATAATAATTTTCTTTGCTGTAATACCTATTCTAATTGGAGCTTTTGGTAATTGGCTTATT

CCTCTATTAGTAGGTGGAAAAGATATAATCTATCCGCGGATAAATAATTTGAGTTATTGGTTATCTCCTAATGCG

CTGTACTTACTTATATTATCTTTTAGAACGGATAAAGGAGTAGGCGCTGGATGGACTATTTACCCGCCACTGTCT

GTATATCCTTATCATAGCGGGCCGAGGATAGATGTTCTTATTGTGTCTTTGCATTTAGCTGGGTTAAGTTCTTTG

GTGGGTGCTATTAATTTTGCTAGTACCAACAAAAACATACCAGTTTTAGAGATAAAAGGAGAACGAGCTGAGCTT

TATGTCCTAAGGATTAGAGTTACTGCCGTATTGCTAATTATTTCTATTCCGGTTTTAATGAGCATATTGTTTGGT

GATAGGTTGTTAAGTGTGGTAGATTTTAGAGAAGTCCTATGTAGCTGG---TTTAAAGCAGGCTTTTTAGTAAAA

AAAGACCTGCTGTTGAGGGGAGTGTGAGATACTTTTCTTTCACATAAAAATTTCATGTTTGGAATAGATGCAGGA

GAT---GGGGGCTTATGCCAGGGAGGAGAAGGTGACGGTGCCCAAGCACGTGTTACTCCTGAAGCTGTATGGGGT

GGTGGAGACACAGCTGTAAATGCGGGCGCCGAAGCCGCTCCTGATAATGCGGAGGGGGCGGGGCGGTACGCTGGA

GACGGCTATGCCCTTCCGTTAGAAGAAGTAGGTTGTAGTTCAGTTGAAGAAAGAGAAAGGGCTGTCGCGGAGCCT

GAAGTTGTATCTAGCGGTTTCGAGCCGGTCGAGCAGAGAGGTGTGCTAATTAGCGAAGCTAGTGGTGCCATTAAT

GCTGGCAAAGAAAGGTTTAGTGATTGT---------------------TAA*

>DL; 'Medu5/1-1001'

'Medu5/1-1001'

CTGATAATCCGGATACAGTTAGGGCATCCTGGAGCAGTCTTTCTAAAAAGAGACTGGTTTTATAATGTGGTTGTT

ACAACACACGCCTTAATGATGATTTTCTTTGCTGTAATACCTATCCTAATCGGAGCTTTTGGTAATTGGCTGATT

CCTCTATTAGTAGGAGGTAAAGATATAATTTATCCGCGGATGAACAATTTGAGATATTGGCTGTCTCCTAACGCG

CTGTACTTACTTATATTATCTTTTAGAACGGATAAAGGGGTAGGTGCTGGATGGACTATTTACCCGCCATTGTCT

GTATACCCTTATCATAGCGGGCCGAGGATAGATGTTCTTATTGTGTCCTTGCATTTAGCTGGGTTAAGTTCTTTG

GTGGGTGCTATTAATTTTGCTAGTACCAACAAAAACATACCAGTTTTAGAGATAAAAGGAGAACGAGCTGAGCTT

TATGTCCTAAGGATTAGAGTTACTGCCGTATTGCTAATTATTTCTATTCCGGTTTTAATGAGCATATTGTTTGGT

GATAGGTTGTTAAGTGTGGTAGATTTTAGAGAAGTCCTATGTAGCTGG---TTTAAAGCAGGCTTTTTAGTAAAA

AAAGACCTGCTGTTGAGGGGAGTGTGAGATACTTTTCTTTCACATAAAAATTCCATGTTTGGAATAGATGCAGGA

GAT---GGGGGCTTATGCCAGGGAGGAGAAGGTGACGGTGCCCAAGTACGTGTTACTCCTGAAGCTGTATGGGGT

GGTGGAGATACACCTGTAAATGCGGGCGCCGAAGCCGCTCCTGATAATGCGGAGGAGGCGGGGCGGTACGTTGGA

GACGGCTATGCCCTTCCGTTAGAAGAAGTAGGTTGTAGTTCAGTTGAAGAAAGAGAAAGGGCTGTCGCGGAGCCT

GAAGTTGTATCTAGCGGTTTCGAGCCGGTCGAGCAGAGAGGTGTGCTAATTAGCGAAGCTAGTGGTGCCATTAAT

GCTGGCAAAGAAAGGTTTAGTGATTGT---------------------TAA*

>DL; 'Medu3/1-1001'

'Medu3/1-1001'

CTGATAATTCGGATACAGTTAGGGCATCCTGGAGCAGTATTTTTAAAAAGAGATTGGTTTTATAATGTGGTTGTT

ACAACACACGCCTTAATAATAATTTTCTTTGCTGTAATACCGATTCTAATCGGAGCTTTTGGTAATTGGCTGATT

CCTCTATTAGTAGGTGGTAAAGATATAATTTATCCGCGGATAAATAATTTGAGTTATTGGTTATCTCCTAATGCG

CTATATTTACTTATATTATCTTTTAGAACGGATAAAGGGGTAGGTGCTGGATGGACTATTTATCCGCCATTGTCT

GTATACCCTTATCATAGCGGGCCGAGGATAGATGTTCTTATTGTGTCGTTGCATTTAGCTGGGTTAAGTTCTTTG

GTGGGTGCTATTAATTTTGCTAGTACCAACAAAAACATACCAGTTTTAGAGATAAAAGGAGAACGAGCTGAGCTT

TATGTCCTAAGGATTAGAGTTACTGCCGTATTGCTAATTATTTCTATTCCGGTTTTAATGAGCATATTGTTTGGT

GATATGTTGTTAAGTGTGGTAGATTTTAGAGAAGTCCTATGTAGCTGG---TTTAAAGCAGGCTTTTTAGTAAAA

AAAGACCTGCTGTTGAGGGGAGTGTGAGATACTTTTCTTTCACATAAAAATTCCATGTTTGGAATAGATGCAGGA

GAT---GGGGGCTTATGCCAGGGAGGAGAAGGTGACGGTGCCCAAGTACGTGTTACTCCTGAAGCTGTATGGGGT

GGTGGAGACACAGCTGCAAATGCGGGCGCCGAAGCCGCTCCTGATAATGCGGAGGGGGCGGGGCGGTACGTTGGA

GACGGCTATGCCCTTCCGTTAGAAGAAGTAGGTTGTAGTTCAGTTGAAGAAAGAGAAAGGGCTGTCGCGGAGCCT

GAAGTTGTATCTAGCGGTTTCGAGCCGGTCGAGCAGAGAGGTGTGCTAATTAGCGAAGCTAGTGGTGCCATTAAT

GCTGGCAAAGAAAGGTTTAGTGATTGT---------------------TAA*

>DL; 'Medu1/1-1001'

'Medu1/1-1001'

CTGATAATTCGGATACAGTTAGGGCATCCTGGAGCAGTATTTTTAAAAAGAGATTGGTTTTATAATGTGGTTGTT

ACAACACACGCCTTAATAATAATTTTCTTTGCTGTAATACCGATTCTAATCGGAGCTTTTGGTAATTGGCTGATT

CCTCTATTAGTAGGTGGTAAAGATATAATTTATCCACGGATAAATAATTTGAGTTATTGGTTATCTCCTAATGCG

CTATATTTACTTATATTATCTTTTAGAACGGATAAAGGGGTAGGTGCTGGATGGACTATTTACCCGCCATTGTCT

GTATATCCTTATCATAGTGGGCCGAGGATAGATGTTCTTATTGTGTCCTTGCATTTAGCTGGGTTAAGTTCTTTG

GTGGGTGCTATTAATTTTGCCAGTACTAACAAAAACATACCAGTTTTAGAGATAAAAGGAGAACGAGCTGAGCTT

TATGTCCTAAGGATTAGAGTTACTGCCGTATTGCTAATTATTTCTATTCCGGTTTTAATGAGCATATTGTTTGGT

GATAGGTTGTTAAGTGTGGTAGATTTTAGAGAAGTCCTATGTAGCTGG---TTTAAAGCAGGCTTTTTAGTAAAA

AAAGACCTACTGTTGAGGGGAGTGTGAGATACTTTTCTTTCACATAAAAATTCCATGTTTGGAATAGATGCAGGA

GAT---GGGGGCTTATGCCAGGGAGGAGAAGGTGACGGTGCCCAAGTACGTGTTACTCCTGAAGCTGTATGGGTT

GGTGGAGACACAGCTGTAAATGCGGGCGCCGAAGCCGCTCCTGATAATGCGGAGGGGGCGGGGCGGTACGTTGGA

GACGGCTATGCCCTTCCGTTAGAAGAAGTAGGTTGTAGTTCAGTTGAAGAAAGAGAAAGGGCTGTCGCGGAGCCT

GAAGTTGTATCTAGCGGTTTCGAGCCGGTCGAGCAGAGAGGTGTGCTAATTAGCGAAGCTAGTGGTGCCATTAAT

GCTGGCAAAGAAAGGTTTAGTGATTGT---------------------TAA*

>DL; 'Medu2/1-1001'

'Medu2/1-1001'

CTGATAATTCGGATACAGTTAGGGCATCCTGGAGCAGTATTTTTAAAAAGAGATTGGTTTTATAATGTGGTTGTT

ACAACACACGCCTTAATAATAATTTTCTTTGCTGTAATACCGATTCTAATCGGAGCTTTTGGTAATTGGCTGATT

CCTCTATTAGTAGGTGGTAAAGATATAATTTATCCACGGATAAATAATTTGAGTTATTGGTTATCTCCTAATGCG

CTATATTTACTTATATTATCTTTTAGAACGGATAAAGGGGTAGGTGCTGGATGGACTATTTACCCGCCATTGTCT

GTATATCCTTATCATAGTGGGCCGAGGATAGATGTTCTTATCGTGTCCTTGCATTTAGCTGGGTTAAGTTCTTTG

GTGGGTGCTATTAATTTTGCCAGTACTAACAAAAACATACCAGTTTTAGAGATAAAAGGAGAACGAGCTGAGCTT

TATGTCCTAAGGATTAGAGTTACTGCCGTATTGCTAATTATTTCTATTCCGGTTTTAATGAGCATATTGTTTGGT

GATATGTTGTTAAGTGTGGTAGATTTTAGAGAAGTCCTATGTAGCTGG---TTTAAAGCAGGCTTTTTAGTAAAA

AAAGACCTGCTGTTGAGGGGAGTGTGAGATACTTTTCTTTCACATAAAAATTCCATGTTTGGAATAGATGCAGGA

GAT---GGGGGCTTATGCCAGGGAGGAGAAGGTGACGGTGCCCAAGTACGTGTTACTCCTGAAGCTGTATGGGGT

GGTGGAGACACAGCTGTAAATGCGGGCGCCGAAGCCGCTCCTGATAATGCGGAGGGGGCGGGGCGGTACGTTGGA

GACGGCTATGCCCTTCCGTTAGAAGAAGTAGGTTGTAGTTCAGTTGAAGAAAGAGAAAGGGCTGTCGCGGAGCCT

GAAGTTGTATCTAGCGGTTTCGAGCCGGTCGAGCAGAGAGGTGTGCTAATTAGCGAAGCTAGTGGTGCCATTAAT

GCTGGCAAAGAAAGGTTTAGTGATTGT---------------------TAA*

>DL; 'Mgal2/1-1001'

'Mgal2/1-1001'

CTGATAATCCGGATACAGTTAGGGCATCCTGGAGCAGTATTTTTAAAAAGAGACTGGTTTTATAATGTGGTTGTT

ACAACACATGCCTTAATAATAATTTTCTTTGCTGTAATACCTATCCTAATCGGAGCTTTTGGTAATTGGCTGATT

CCCCTATTAGTAGGTGGTAAAGATATAATTTATCCGCGGATAAATAATTTGAGTTATTGGTTATCTCCTAATGCG

CTATATTTACTTATATTATCTTTTAGAACGGATAAAGGGGTAGGTGCTGGATGGACTATTTACCCGCCATTGTCT

GTATACCCTTATCATAGCGGGCCGAGGATAGATGTTCTTATTGTGTCCTTGCATTTAGCTGGGTTAAGTTCTTTG

GTGGGTGCTATTAATTTTGCTAGTACCAACAAAAACATACCAGTTTTAGAGATAAAAGGAGAACGAGCTGAGCTT

TATGTCCTAAGGATTAGAGTTACTGCCGTATTGCTAATTATTTCTATTCCGGTTTTAATGAGCATATTGTTTGGT

GATAGGTTGTTAAGTGTGGTAGATTTTAGAGAAGTCCTATGTAGCTGG---TTTAAAGCAGGCTTTTTAGTAAAA

AAAGACCTGCTGTTGAGGGGAGTGTGAGATACTTTTCTTTCACATAAAAATTCCATGTTTGGAATAGATGCAGGA

GAT---GGGGGCTTATGCCAGGGAGGAGAAGGTGACGGTGCCCAAGTACGTGTTACTCCTGAAGCTGTATGGGGT

GGTGGAGATACACCTGTAAATGCGGGCGCCGAAGCCGCTCCTGATAATGCGGAGGAGGCGGGGCGGTACGTTGGA

GACGGCTATGCCCTTCCGCTAGAAGAAGTAGGTTGTAGTTCAGTTGAAGAAAGAGAAAGGGCTGTCGCGGAGCCT

GAAGTTGTATCTAGCGGTTTCGAGCCGGTCGAGCAGAGAGGTGTGCTAATTAGCGAAGCTAGTGGTGCCATTAAT

GCTGGCAAAGAAAGGTTTAGTGATTGT---------------------TAA*

>DL; 'Medu4/1-1001'

'Medu4/1-1001'

CTGATAATTCGGATACAGTTAGGGCATCCTGGAGCAGTATTTTTAAAAAGAGATTGGTTTTATAATGTGGTTGTT

ACAACACACGCCTTAATAATAATTTTTTTTGCTGTAATACCCATTCTAATCGGAGCTTTTGGTAATTGGCTGATT

CCTCTATTAGTAGGTGGTAAAGATATAATTTATCCGCGGATAAATAATTTGAGCTATTGGTTATCTCCTAATGCG

CTATATTTACTTATATTATCTTTTAGAACGGATAAAGGGGTAGGTGCTGGATGGACTATTTACCCACCATTGTCT

GTATATCCTTATCATAGCGGGCCGAGGATAGATGTTCTTATTGTGTCTTTGCATTTAGCTGGGTTAAGTTCTTTG

GTGGGTGCTATTAATTTTGCTAGTACCAACAAAAACATACCAGTTTTAGAGATAAAAGGAGAACGAGCTGAGCTT

TATGTCCTAAGGATTAGAGTTACTGCCGTACTGCTAATTATTTCTATTCCGGTTTTAATGAGCATATTGTTTGGT

GATAGGTTGTTAAGTGTGGTAGATTTTAGAGAAGTCCTATGTAGCTGG---TTTAAAGCAGGCTTTTTAGTAAAA

AAAGACCTGCTGTTGAGGGGAGTGTGAGATACTTTTCTTTCACATAAAAATTCCATGTTTGGAATAGATGCAGGA

GAT---GGGGGCTTATGCCAGGGAGGAGAAGGTGACGGTGCCCAAGTACGTGTTACTCCTGAAGCTGTATGGGAT

GGTGGAGATACACCTGTAAATGCGGGCGCCGAAGCCGCTCCTGATAATGCGGAGGAGGCGGGGCGGTACGTTGGA

GACGGCTATGCCCTTCCGTTAGAAGAAGTAGGTTGTAGTTCAGTTGAAGAAAGAGAAAGGGCTGTCGCGGAGCCT

GAAGTTGTATCTAGCGGTTTCGAGCCGGTCGAGCAGAGAGGTGTGCTAATTAGCGAAGCTAGTGGTGCCATTAAT

GCTGGCAAAGAAAGGTTTAGTGATTGT---------------------TAA*

**Bayesian Ka/Ks ratios for the MEC model (F-ORF starting at position 170)**

**
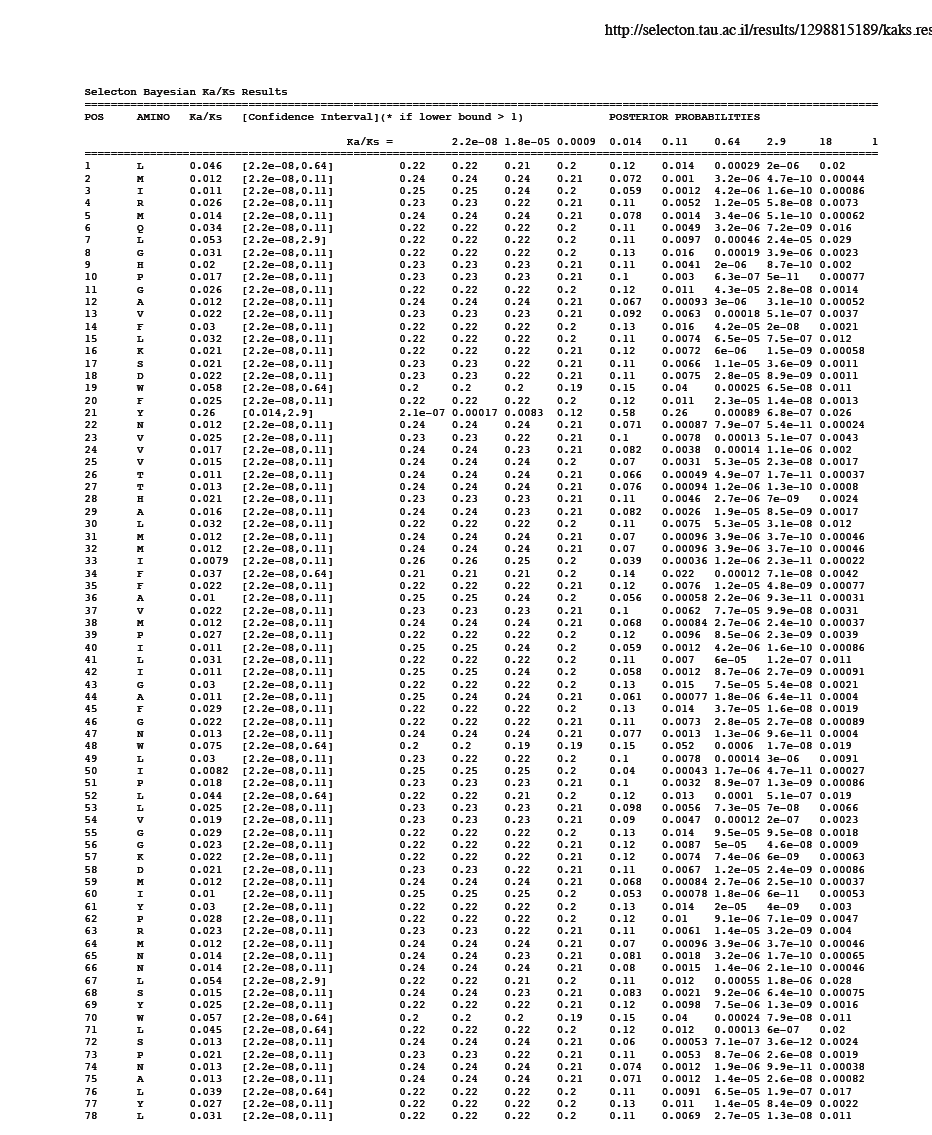
**

**
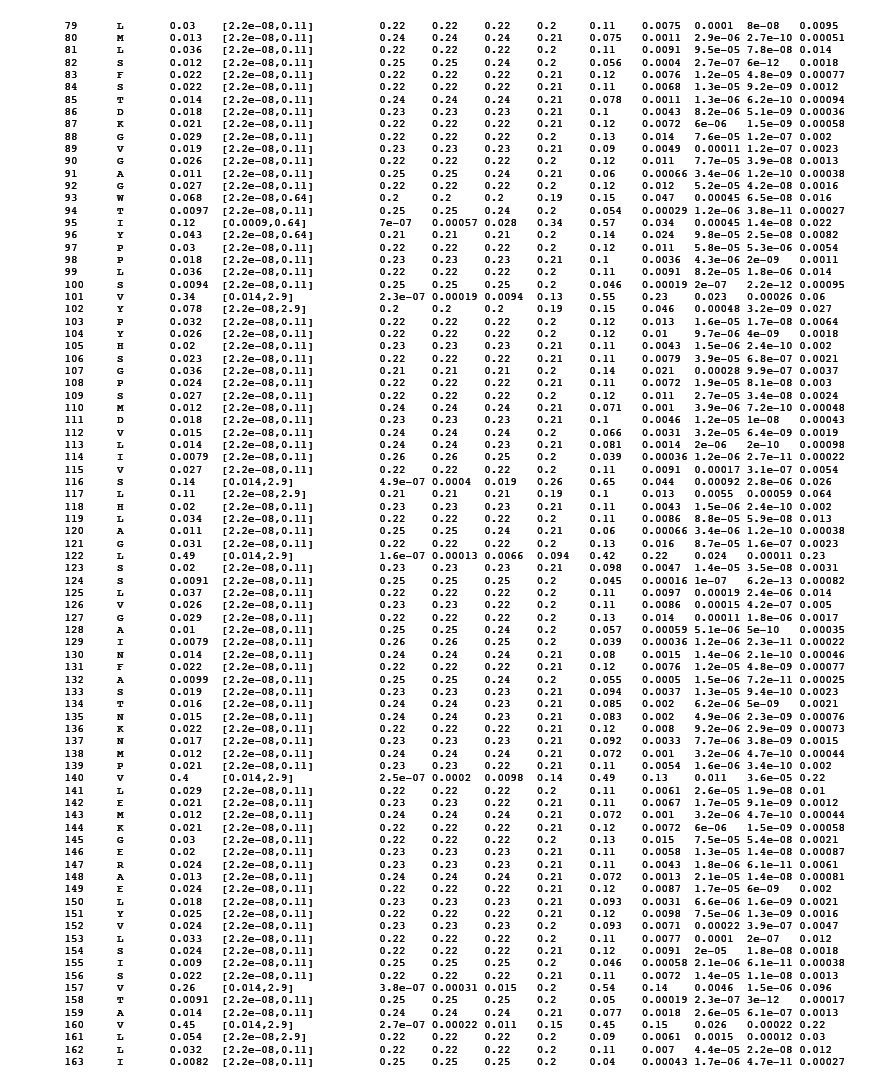
**

**
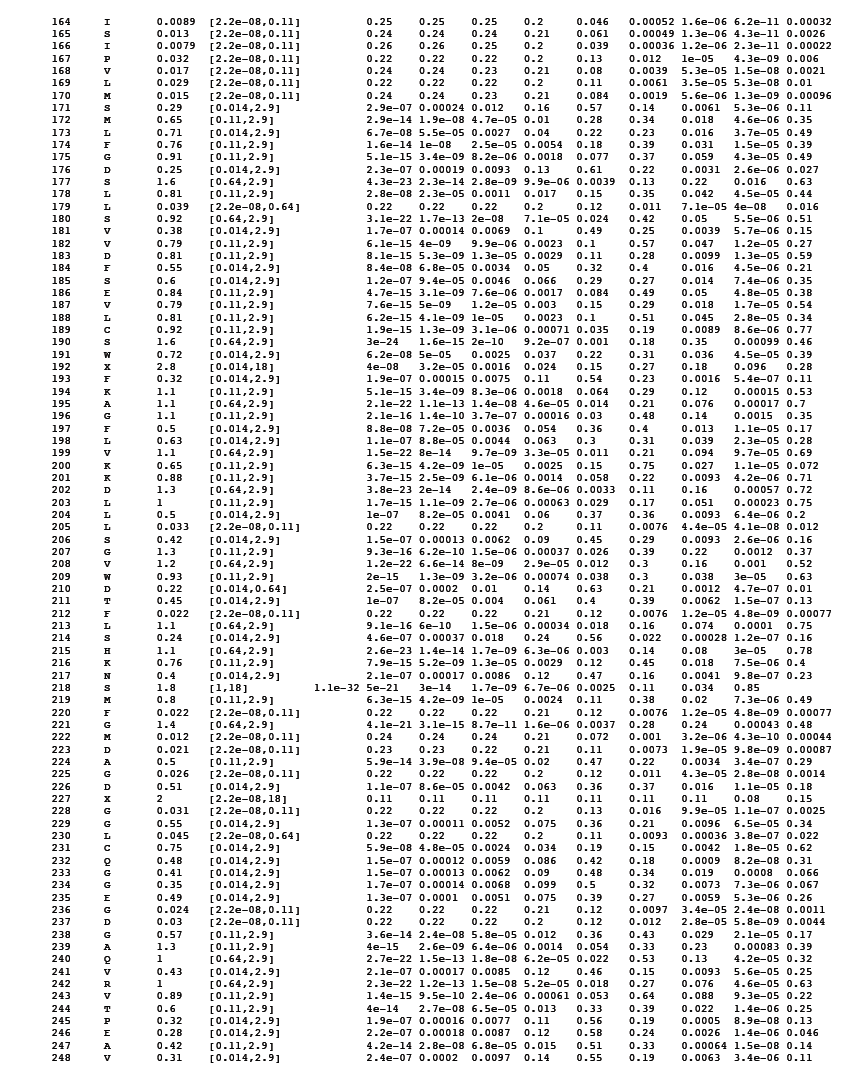
**

**
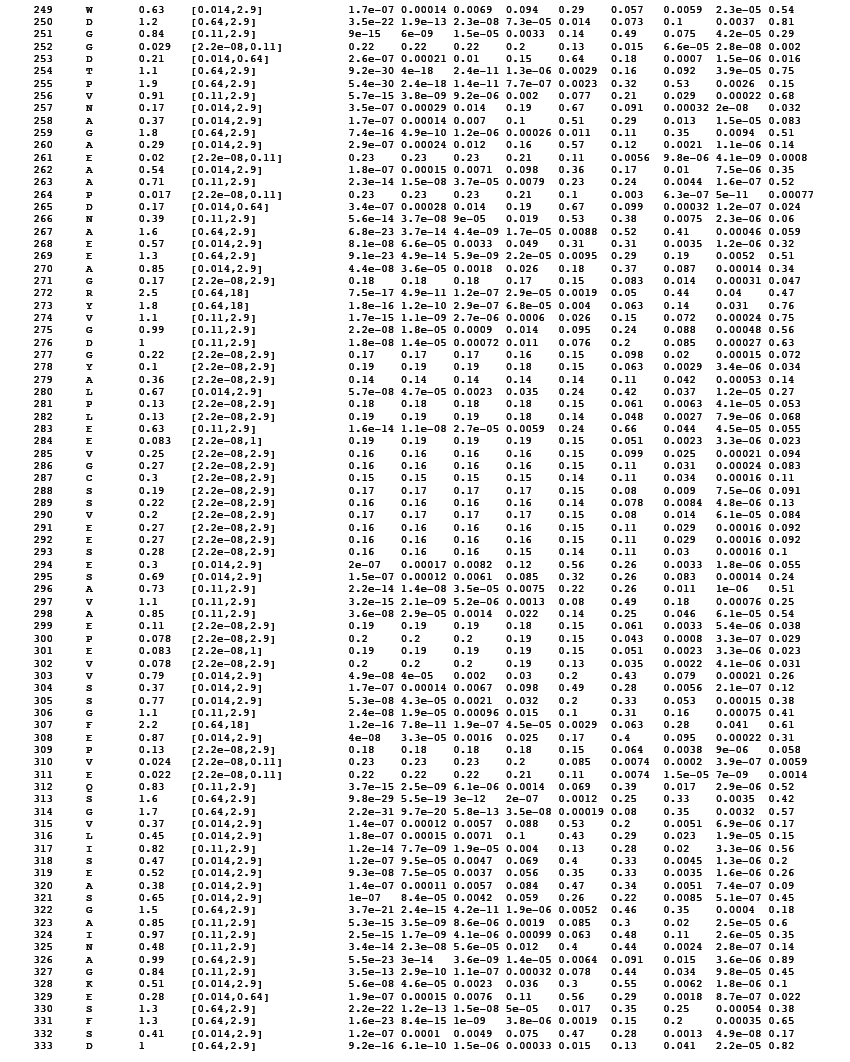
**

**
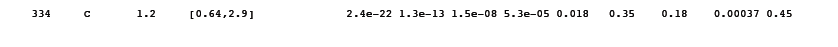
**
