# Supplementary figures and images for: Evidence for a Fourteenth mtDNA-Encoded Protein in the Female-Transmitted mtDNA of Marine Mussels (Bivalvia: Mytilidae)
Source: PLoS One. 2011 Apr 27;6(4):e19365. doi: 10.1371/journal.pone.0019365 (PMC3083442; doi:10.1371/journal.pone.0019365)

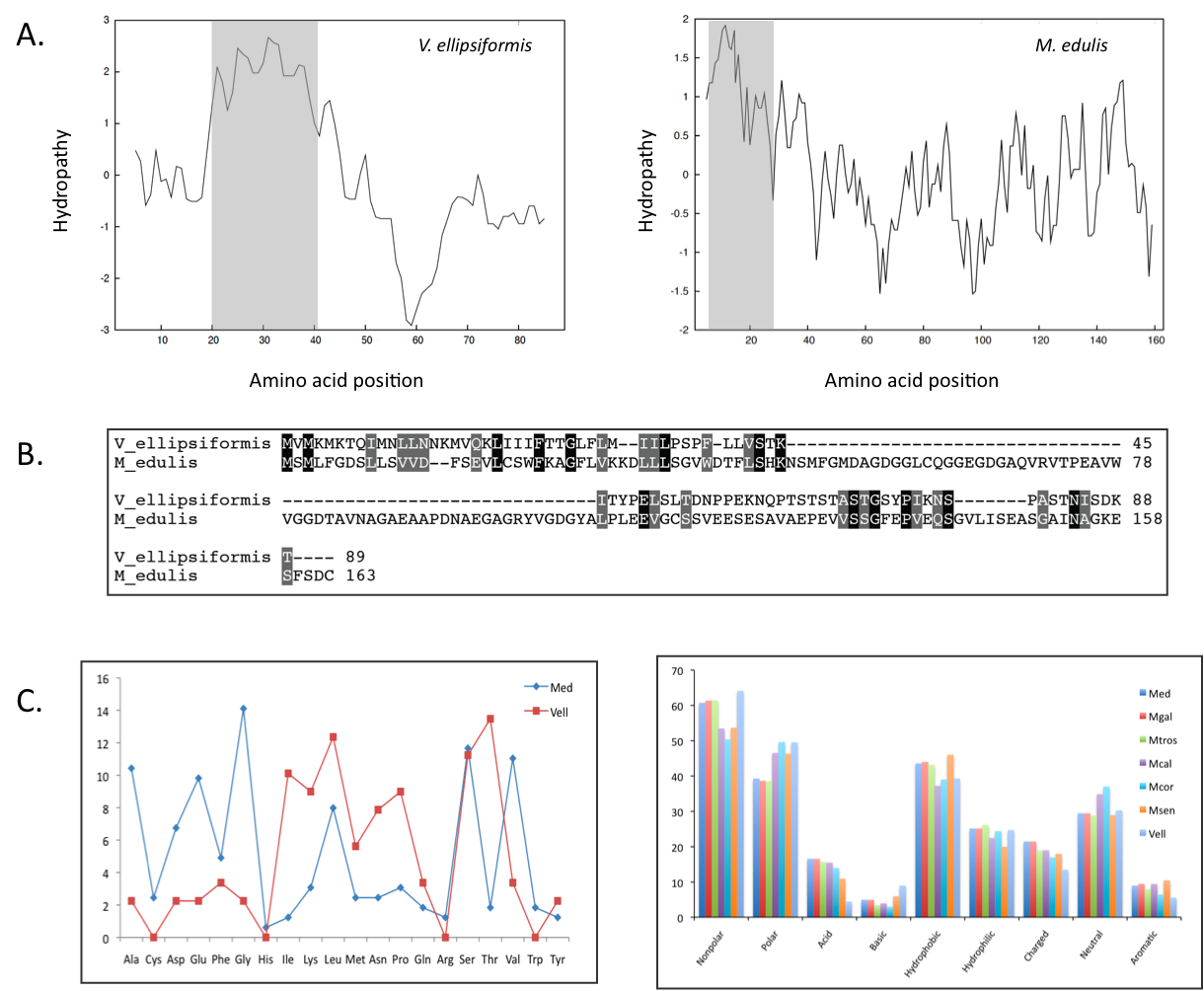

Supplement: Figure S4 — (A) Comparisons of Mytilus edulis F- ORF-VD1 and Venustaconcha ellipsiformis F- ORF hydropathy profiles. Profiles were calculated by the method of Kyte and Doolittle [104]. Numbers below profiles designate amino acid positions in each protein. Predicted transmembrane domains according to TMpred [102] are shown in light gray. (B) Alignment of the translated F-orf-vd1 M. edulis and F-orf V. ellipsiformis sequences. Identical amino acids are highlighted in black. Chemically equivalent amino acids are in gray. Dashes (–) denote a missing residue at this position in comparison with other sequence(s). (C) Overall amino acid composition (left) and composition of chemically equivalent amino acids (right) of Mytilus spp. F-ORF-VD1, M. senhousia F-ORF and V. ellipsiformis F-ORF protein sequences. Amino acid composition is reported as percentage. (TIF) [file pone.0019365.s004.tif]
